# Theoretical insights into rolicyclidine adsorption and sensing using modified C_60_ fullerene

**DOI:** 10.3389/fchem.2026.1908245

**Published:** 2026-10-05

**Authors:** Bader Huwaimel, Saad Alqarni, Ali Alghubayshi, Talal Alotaibi, Hashem O. Alsaab, Rami M. Alzhrani, Amr S. Abouzied

**Affiliations:** 1 Department of Pharmaceutical Chemistry, College of Pharmacy, University of Ha’il, Ha’il, Saudi Arabia; 2 Medical and Diagnostic Research Center, University of Ha’il, Hail, Saudi Arabia; 3 Department of Clinical Pharmacy, College of Pharmacy, University of Hail, Hail, Saudi Arabia; 4 Department of Pharmaceutics and Industrial Pharmacy, College of Pharmacy, Taif University, Taif, Saudi Arabia

**Keywords:** 1-(1-Phenylcyclohexyl)pyrrolidine, adsorption, drug detection, electrochemical sensor, fullerene C_60_, rolicyclidine

## Abstract

1-(1-Phenylcyclohexyl) pyrrolidine (Rolicyclidine (Php)) is a potent dissociative arylcyclohexylamine with psychotropic properties, and finding methods for rapid, accurate, and facile detection of Php by traditional analytical methods remains an ongoing challenge. To address this challenge, in this study, the structural, electronic, and adsorption properties of pristine and boron- and silicon-doped C_60_ fullerenes (BC_59_ and SiC_59_, respectively) were investigated using DFT theory to identify suitable candidates for Php adsorption and detection. The robustness of the predicted trends was assessed using the B97D/6–31G(d), ωB97XD/6–31G(d), and B97D/6–311G (d,p) computational levels, while selected calculated properties of pristine C_60_ were compared with available experimental data. The calculations suggest that B and Si substitution modify the structural and electronic properties of pristine C_60_, with Si substitution producing the largest local structural distortion. The BSSE-corrected adsorption energies indicate relatively weak-to-moderate Php adsorption on C_60_ and BC_59_, whereas SiC_59_ is predicted to provide substantially stronger adsorption. At the B97D/6–31G(d) level, the corrected adsorption energies were −6.96, −8.04, and −38.79 kcal.mol^−1^ for C_60_@Php, BC_59_@Php, and SiC_59_@Php, respectively. The additional B97D/6–311G (d,p) calculations produced the same qualitative adsorption trend, confirming that the main conclusions are not dependent on the selected basis set. Based on this comparative theoretical screening, SiC59 may be prioritized for experimental investigation as a strong Php-adsorption material, whereas BC59 may be prioritized as a candidate sensing material because of its predicted reversibility and comparatively large adsorption-induced electronic perturbation. These assignments represent theoretical screening results and require validation through material fabrication, selectivity testing, kinetic measurements, and evaluation under realistic operating conditions.

## 1 Introduction

1-(1-Phenylcyclohexyl) pyrrolidine, commonly known as rolicyclidine (Php), is an arylcyclohexylamine dissociative compound structurally related to phencyclidine (PCP). Acting primarily as an N-methyl-D-aspartate (NMDA) receptor antagonist, Php can produce anesthetic, hallucinogenic, and neurobehavioral effects. Its recreational misuse may be accompanied by hallucinations, agitation, paranoia, impaired motor function, hypertension, and respiratory depression. Therefore, reliable identification of Php in biological and forensic samples is important for clinical toxicology, public health, and law enforcement applications (Morris and Wallach, 2014; Quinn et al., 2020; Vaillancourt et al., 2021). Conventional analytical techniques, including gas chromatography-mass spectrometry (GC-MS), liquid chromatography-mass spectrometry (LC-MS), high-performance liquid chromatography (HPLC), and infrared spectroscopy, can provide accurate identification. Nevertheless, these methods commonly require specialized instrumentation, trained personnel, laboratory facilities, and relatively extensive sample preparation, limiting their applicability to rapid or field-based screening (Baker and Phillips, 1983; Kuchinos, 2022).

Carbon-based nanomaterials, including carbon nanotubes, graphene derivatives, and fullerenes, have attracted considerable attention as potential sensing platforms because their electronic properties can be measurably perturbed through interactions with molecular analytes (Guliy and Dykman, 2025; Rocha et al., 2025). Among these materials, the C_60_ fullerene possesses a highly conjugated, curved carbon framework, a large accessible surface area, favorable electron-accepting characteristics, and considerable chemical stability (David et al., 1992; Pan et al., 2020). These features facilitate noncovalent interactions, including dispersion forces, π-π interactions, and donor-acceptor interactions, making C_60_ and its derivatives promising candidates for molecular adsorption and sensing applications.

Several computational investigations have evaluated fullerene-based materials for pharmaceutical and drug-related applications. Extrinsically modified C_60_ fullerenes have been studied as work-function-type sensors for amphetamine (Bashiri et al., 2017), while C_60_ has also been investigated as a carrier for anticancer agents (Kumar and Raza, 2017). Parlak and Alver examined single-, double-, and triple-boron-modified C_60_ structures for the delivery and sensing of tegafur (Parlak and Alver, 2025). Functionalized C_60_ nanocages have also been evaluated as adsorbents and sensors for methamphetamine (Hadi et al., 2024a). In another investigation, pristine C_60_ and Si- and Al-doped B_12_N_12_ nanocages were examined as potential sensing materials for dacarbazine (Maleki et al., 2024). Collectively, these studies indicate that structural modification and heteroatom incorporation can alter the adsorption affinity, charge-transfer behavior, and electronic response of fullerene-based systems.

Computational chemistry, especially density functional theory (DFT), provides an efficient pre-experimental framework for comparing molecular geometries, adsorption energies, electronic structures, and charge-transfer processes, and ultimately for selecting suitable candidates for further experimental evaluation (Goh et al., 2017). Computational methods have also been applied to bioactive compounds related to the central nervous system; for example, Todkar et al. combined *in silico* screening and DFT calculations to investigate *Nelumbo nucifera* phytochemicals as potential β-site amyloid precursor protein-cleaving enzyme 1 (BACE-1) inhibitors for Alzheimer’s disease (Todkar et al., 2025). This research demonstrates the value of computational approaches for elucidating the molecular-level behavior of biologically active compounds prior to experimental evaluation and for identifying superior candidates.

Recent reviews have provided broader insight into the structural and electronic consequences of heteroatom incorporation into fullerene frameworks. Zielińska-Pisklak et al. reviewed germanium-containing carbon fullerenes and summarized quantum-chemical findings concerning substitution, adsorption, and endohedral or exohedral encapsulation (Zielińska-Pisklak et al., 2025a). The same research group also comprehensively reviewed substitutional Si-doped C_60_ systems, including their synthetic strategies, structural characteristics, electronic properties, and molecular-modeling approaches (Zielińska-Pisklak et al., 2025b). These reviews demonstrate that heteroatom substitution can break the symmetry of the original fullerene cage, redistribute its electronic density, introduce localized reactive sites, and modify its interactions with guest molecules.

The practical and theoretical relevance of B- and Si-modified fullerenes is further supported by previous studies. Arie and Lee experimentally investigated boron-doped C_60_ films and demonstrated the feasibility of preparing electrochemically active boron-containing fullerene materials (Arie and Lee, 2011). Yadav et al. theoretically demonstrated that B and Si substitutions modify the adsorption and sensing behavior of C_60_ toward epinephrine (Yadav et al., 2025). Parlak et al. investigated the interaction of molnupiravir with Si-doped C_60_ and reported substantial adsorption-induced changes in the electronic properties of the modified fullerene (Parlak et al., 2021). In addition, Shariatinia and Kurban theoretically examined functionalized (B,N)-C_60_ systems and demonstrated that dopant incorporation and surface functionalization can tune their electron-transport characteristics (Shariatinia and Kurban, 2024). These findings support the selection of boron and silicon as suitable dopants to modify the molecular recognition and electronic response properties of C_60_.

Despite these advances, the adsorption and sensing behavior of Php on pristine C_60_ and its substitutionally B- and Si-doped derivatives has not yet been systematically compared. Therefore, the present study employs DFT calculations (Orio et al., 2009) to investigate the interactions of Php with C_60_, BC_59_, and SiC_59_. Structural deformation, adsorption energy, Frontier molecular orbitals, electronic reactivity, charge transfer, recovery time, conductivity-related response, and the nature of the intermolecular interactions are comparatively evaluated. Two dispersion-inclusive functionals are employed to assess the consistency of the predicted trends. Particular attention is given to distinguishing adsorption performance from sensing performance so that structures suitable for strong Php capture can be differentiated from those capable of producing a reversible and detectable electronic response.

## 2 Computational section

In the first step, the molecular structures of Php, C_60_, BC_59,_ and SiC_59_ (as shown in Figure 1) were studied. Initial geometries of isolated Php, C_60_, BC_59_, and SiC_59_, as well as the corresponding 1:1 C_60_@Php, BC_59_@Php, and SiC_59_@Php complexes, were constructed using GaussView 6.0. All geometries were subsequently optimized using Gaussian 09W (Frisc and h, 2009). The optimized energy profiles for each of the designed structures are reported in Supplementary Figure S1.

**FIGURE 1:**
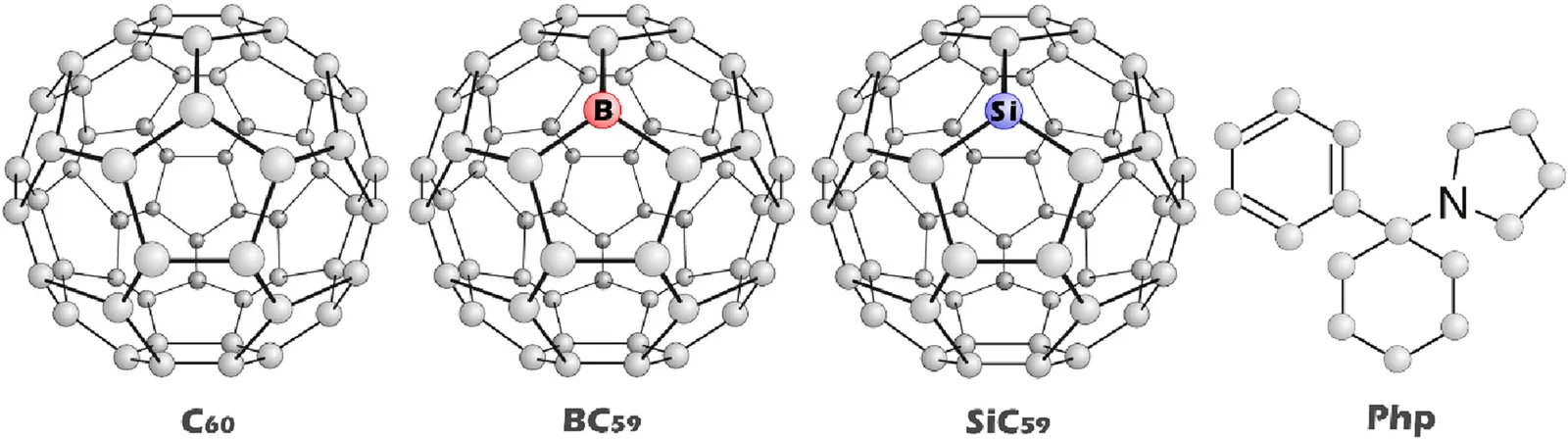
Molecular structure of Php, pristine C_60_, and its B- and Si-doped forms (BC59 and SiC_59_).

The geometry optimizations were initially performed in the water phase using the B97D/6-31G(d) level of theory with the CPCM solvation model. The 6-31G(d) basis set was selected as a polarized split-valence basis set that provides a reasonable balance between computational cost and accuracy for the relatively large fullerene-drug complexes considered here. The inclusion of polarization functions is important for describing charge redistribution, dopant-site polarization, and non-covalent adsorption interactions. To assess possible basis-set dependence, all key calculations were also repeated at the larger B97D/6-311G (d,p) level. Although the absolute numerical values changed to some extent, the same qualitative trends were obtained for the electronic descriptors and adsorption parameters, confirming that the main conclusions are not artifacts of the smaller basis set. The B97D functional was selected because it includes Grimme’s empirical dispersion correction, making it suitable for fullerene-based adsorption systems where dispersion, π-π contacts, van der Waals forces, and weak donor–acceptor interactions play major roles. In addition, the ωB97XD functional was used as an independent dispersion-corrected range-separated hybrid functional to further validate the trends (Avramopoulos et al., 2016; Ovalle and Malardier-Jugroot, 2022). Because ωB97XD includes range-separated exact exchange whereas B97D is a semilocal functional without exact exchange, larger absolute Kohn-Sham HOMO-LUMO gaps are expected with ωB97XD. Accordingly, the two functionals were used primarily to assess the robustness of relative trends and structural rankings rather than to obtain numerically identical orbital gaps. The agreement among B97D/6-31G(d), B97D/6-311G (d,p), and ωB97XD/6-31G(d) supports the use of the selected theoretical levels for comparative analysis of the relative adsorption and electronic-response trends.

Water was selected as the continuum solvent because Php detection may be performed in aqueous biological or forensic matrices. The CPCM approach accounts for the bulk dielectric polarization of water and its effects on molecular geometry, charge distribution, and noncovalent interactions. However, it does not explicitly represent individual water molecules, specific solute-solvent hydrogen bonds, ionic strength, pH, or the complex components present in serum, urine, and oral fluid. Therefore, the CPCM calculations provide an approximate aqueous-phase description suitable for comparative screening but should not be considered a complete representation of realistic biological matrices (Samavat et al., 2021).

To evaluate the sensitivity of the calculated results to the bulk dielectric response of a physiological aqueous medium, an additional set of CPCM single-point calculations was performed at the B97D/6-31G(d) level using a user-defined relative dielectric constant of εr = 76.2. This value was adopted as an approximate representation of dielectric screening in a physiological-saline-like aqueous NaCl medium at 298 K, rather than as a complete model of blood, plasma, or serum (Buchner et al., 1999; Chelidze, 2002). The calculations were performed on the previously optimized water-phase geometries using the Gaussian keyword SCRF=(CPCM, Read), followed by the specification εr = 76.2. The isolated Php molecule, the isolated C_60_, BC_59_, and SiC_59_ structures, and their corresponding 1:1 complexes were recalculated consistently using the same dielectric constant. The adsorption energies were obtained from the complex and isolated-component energies evaluated in the same continuum medium and were corrected for basis-set superposition error using the counterpoise method. The resulting adsorption energies, recovery-time estimates, and gap-derived conductivity descriptors were compared with the corresponding CPCM-water results to determine whether the predicted trends were sensitive to the selected dielectric constant. The εr = 76.2 calculations account only for an average bulk dielectric-screening effect. They do not explicitly include proteins, buffer components, individual ions, specific solute–solvent interactions, pH-dependent protonation, hematocrit, or other constituents of real blood and biological samples. Furthermore, because the present molecular models contain one Php molecule and one fullerene cage, they represent 1:1 molecular complexes rather than a prescribed bulk analyte concentration. Consequently, these calculations cannot directly determine aqueous solubility, concentration-dependent response, detection limits, or sensing performance below an IC50 threshold. The experimental aqueous solubility of Php was not determined in the present study, and no quantitative solubility or concentration value can be inferred from the continuum-solvation calculations or the investigated 1:1 molecular complexes. Such properties require concentration-dependent experimental evaluation under realistic biological-matrix conditions.

Frequency calculations were also performed, and the absence of imaginary frequencies confirmed that all structures are stable and not in a transition state. To ensure the robustness and consistency of the reported results, all calculations were repeated using the ωB97XD functional while maintaining the same basis set and solvent model (water phase). In DFT studies, computed structural and energetic parameters can be sensitive to the choice of exchange-correlation functional. Therefore, re-evaluating the systems with an alternative functional allows verification that the observed trends and conclusions are not artifacts of a particular methodological choice. The ωB97XD functional is a range-separated hybrid functional that includes empirical dispersion corrections. Its long-range treatment of exchange interactions and explicit description of dispersion effects provide a reliable account of noncovalent and charge-transfer interactions, which are particularly relevant in solvated systems. Consequently, its use offers an additional level of validation and strengthens confidence in the reliability of the computational results (Chai and Head-Gordon, 2008).

Reactivity parameters including: energy gap (HLG), chemical softness (S), chemical hardness (η), chemical potential (μ), maximum possible charge transfer (ΔNmax) and electrophilicity-based charge transfer (ECT) were evaluated using Equations 1–6 (Padmanabhan et al., 2007; Pearson, 2005).

$$\text{HLG}=\left|E_{\text{HOMO}}-E_{\text{LUMO}}\right|$$ (1)

$$\eta =\left(-E_{\text{HOMO}}-\left(-E_{\text{LUMO}}\;\right)\right)/2$$ (2)

$$\mu =-\left(-E_{\text{HOMO}}+\left(-E_{\text{LUMO}}\right)\right)/2$$ (3)

S=1/2η (4)

ΔNmax=−μη (5)

$$ECT={\left({\Delta N}_{\max}\right)}_{\alpha }-{\left({\Delta N}_{\max}\right)}_{\beta }$$ (6)

In Equations 1–6, E_HOMO_ is the energy of the highest orbital occupied by electrons, and E_LUMO_ is the energy of the lowest unoccupied orbital by electrons (Hadi et al., 2024b). Additionally, α is associated with the isolated fullerene-based structure (C_60_, BC_59_, and SiC_59_), and β pertains to the complex of the Surface@Php (C_60_@Php, BC_59_@Php, and SiC_59_@Php). If the ECT value is negative, the fullerene-based structure exhibits an electron-accepting tendency, suggesting electron transfer from Php toward the fullerene-based structure, and *vice versa* (Hadi et al., 2024b).

The most important parameters related to the evaluation of sensing properties, including adsorption energy (Eads), recovery time (τ), and electrical conductivity (σ), were computationally evaluated using Equations 7–9, respectively.

$$E_{\text{ads}}=E_{\left(\text{Si}/B\right)C60@\text{Drug}}-\left(E_{Drug}+E_{\left(\text{Si}/B\right)C60}\right)+E_{BSSE}$$ (7)

$$\tau =V_{0}^{-1}\times \exp\left(-\frac{E_{ads}}{k_{B}T}\right)$$ (8)

$$\sigma =AT^{3/2}e^{\left(-HLG/2K_{B}T\right)}$$ (9)

In Equation 7, E ((Si/B)C_60_@Drug) is the energy of each fullerene-Php complex (C_60_, BC_59_, and SiC_59_) with Php. E_Php_ and E_(Si/B)C60_ represent the energy of Php and that of each isolated fullerene-based structure, respectively. Basis set superposition error (BSSE) was calculated using the counterpoise correction method (using the Counterpoise = 2 keyword). Each optimized complex was divided into two fragments: the fullerene-based adsorbent and Php. The fragment energies were recalculated in the full complex basis set using ghost atoms, and the resulting BSSE value was used to obtain the corrected adsorption energy (Kestner and Combariza, 1999).

In Equation 8, ν_0_ denotes the attempt-frequency prefactor. To enable comparison with previous theoretical adsorption and sensing studies, the recovery time was first estimated using the conventional fixed value of ν0 = 10^12^ s^1^. In addition, system-specific attempt frequencies were obtained from the B97D/6–31G(d) harmonic vibrational outputs evaluated at the CPCM-water-optimized geometries of the C60@Php, BC59@Php, and SiC59@Php complexes. The low-frequency normal-coordinate displacement vectors were examined, and the modes predominantly associated with the relative stretching/translation of Php with respect to the fullerene surface along the adsorption–desorption coordinate were selected. The corresponding wavenumbers were converted to ordinary frequencies using ν_0_,vib = cν, where c = 2.99792458 × 10^10^ cm s^−1^. The selected wavenumbers were 48.5883, 38.0897, and 95.0201 cm^−1^ for C_60_@Php, BC_59_@Php, and SiC_59_@Php, respectively, corresponding to system-specific attempt frequencies of 1.4566 × 10^12^, 1.1419 × 10^12^, and 2.8486 × 10^12^ s^−1^. Both the conventional fixed-prefactor recovery times and the vibrationally derived recovery times were retained to evaluate the sensitivity of the results to the selected attempt-frequency treatment. Because the magnitude of the BSSE-corrected adsorption energy was used as an approximation to the desorption barrier, both sets of recovery times should be interpreted as comparative theoretical estimates rather than experimentally measured kinetic lifetimes.

In Equations 8, 9, k_B_ is the Boltzmann constant and T is the temperature, which was set to 298 K. In Equation 9, A is the Richardson constant, with a value of 6 × 10^5^ A.m^−2^. K^−2^ (Liaqat et al., 2025; Rezaei et al., 2020).

To quantify the internal consistency among the selected computational levels, mean absolute deviation, root-mean-square deviation, and Pearson correlation coefficients were calculated for the principal descriptors. The Pearson coefficients indicate that the relative structural trends are generally preserved, whereas the MAD and RMSD values demonstrate that some absolute quantities, particularly the Kohn-Sham orbital gaps, remain method-dependent. These statistical comparisons assess agreement among the computational datasets and should not be interpreted as experimental validation. For reproducibility, the XYZ coordinates of each designed structure are reported in Supplementary Table S1.

The calculated descriptors should be interpreted as comparative theoretical indicators rather than direct substitutes for experimentally measured sensor characteristics. In particular, changes in the HOMO-LUMO gap, softness, charge-transfer descriptors, DOS/PDOS profiles, and gap-derived conductivity estimates cannot independently establish sensitivity, selectivity, detection limit, response time, or overall device performance. In this study, these parameters are used only to compare adsorption-induced electronic perturbations among C_60_, BC_59_, and SiC_59_ and to identify candidates for subsequent experimental investigation. Practical sensing performance must be evaluated after material synthesis and electrode fabrication and under relevant solvent, matrix, kinetic, interference, and operating conditions.

## 3 Results and discussion

### 3.1 Bond length and bond angle

In the context of molecular design, it is important to study bond lengths and bond angles, as these parameters are key determinants of a molecule’s geometry, stability, and electronic properties, all of which influence the molecule’s reactivity and interactions with surrounding species. Doping can have a large impact on these structural parameters, since a dopant may introduce atoms of different sizes and electronegativities, thereby altering the local electronic distribution and bonding environment of the pristine molecule. This can create small distortions in the overall molecular geometry; however, even a small distortion can enhance charge transfer, adsorption capabilities, and overall sensing performance of the doped structure (Shirley et al., 1995). For this purpose, bond lengths and angles between relevant atoms (see Figure 2) in pristine C_60_ and the doped versions, BC_59_ and SiC_59_, were calculated, and the results are listed in Table 1.

**FIGURE 2:**
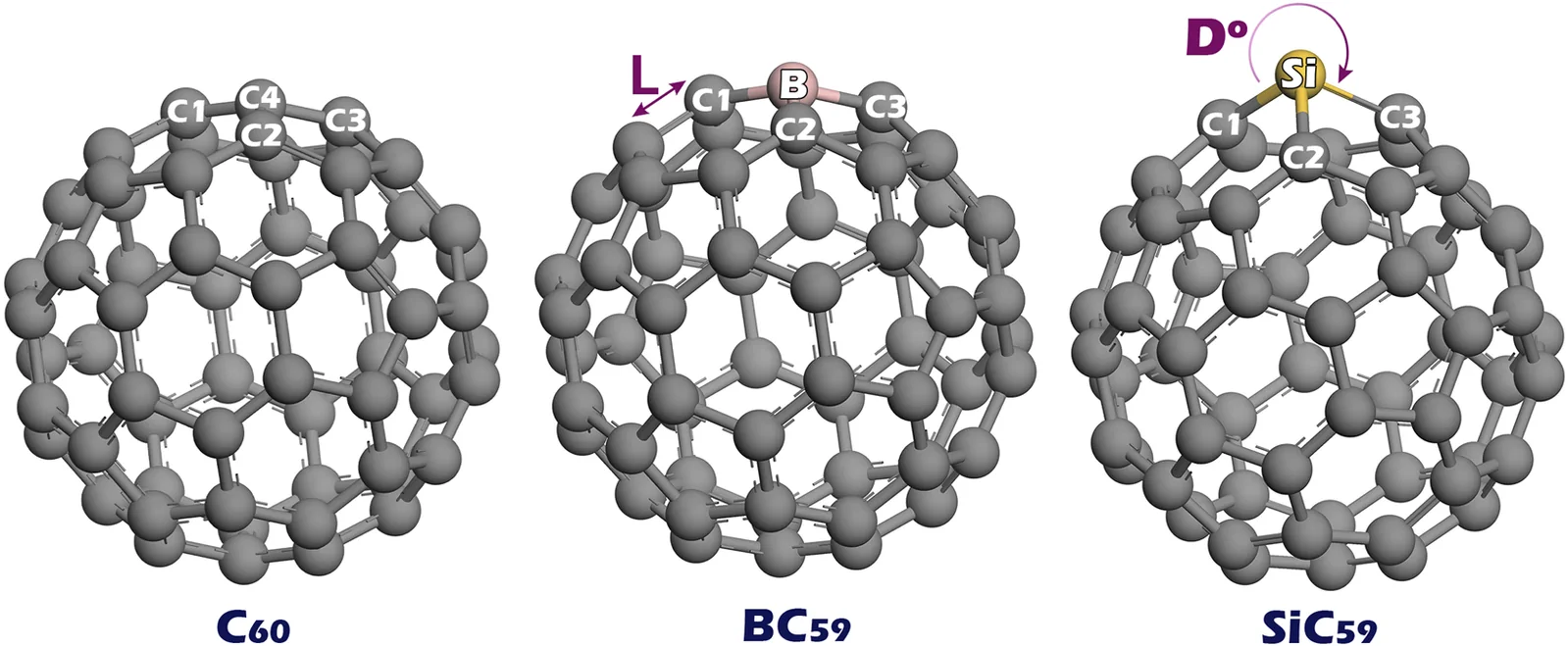
Optimized structure of each of the structures designed in this work.

**TABLE 1:** Values of bond length (L) and bond angles (D) between some important atoms in the studied fullerene structures (C_60_, BC_59_ and SiC_59_).

Structure	Bond lengths (Å)			Bond angles (°)	
	​	Theoretical	Experimental		
C_60_	C4-C1	1.45	1.40 to 1.45 (David et al., 1991)	C1-C4-C2	120.01
	C4-C2	1.40		C1-C4-C3	108.00
	C4-C3	1.45		C2-C4-C3	119.99
BC_59_	B-C1	1.53	---------	C1-B-C2	118.46
	B-C2	1.55	---------	C1-B-C3	118.46
	B-C3	1.55	---------	C2-B-C3	106.26
SiC_59_	Si-C1	1.81	---------	C1-Si-C2	99.47
	Si-C2	1.86	---------	C1-Si-C3	99.47
	Si-C3	1.86	---------	C2-Si-C4	90.37

In pristine C_60_, the C-C bond lengths (C4-C1, C4-C2, and C4-C3) are 1.455, 1.404, and 1.455 Å, respectively. This slight alteration in bond lengths reflects the intrinsic curvature of the fullerene cage and is consistent with a predominantly sp^2^-hybridized carbon network. The corresponding bond angles (C1-C4-C2, C1-C4-C3, and C2-C4-C3) range from 108° to 120°, which mirrors the geometric balance between pentagonal and hexagonal sites within the molecular framework. This configuration maintains a high degree of structural symmetry and minimizes internal strain, thereby contributing to the remarkable stability of the undoped fullerene structure.

When boron is introduced into the cage to form BC_59_, the geometry undergoes measurable but moderate perturbation. The B-C bond lengths increase slightly to 1.533–1.555 Å, reflecting boron’s larger covalent radius and the weaker B-C bond compared to C-C. The bond angles around the dopant (C1-B-C2, C1-B-C3, and C2-B-C3) range from 106.26° to 118.46°, indicating mild angular compression relative to the pristine structure. The hybridization around boron remains close to sp^2^, as shown by the two nearly planar angles near 120° and one slightly compressed angle accommodating the curvature of the cage. These modifications suggest that boron doping preserves the fundamental electronic and structural characteristics of the fullerene while inducing a controlled degree of local distortion.

More significant deviations are observed upon silicon doping to produce SiC_59_. The Si-C bonds elongate substantially to 1.81–1.86 Å, consistent with the larger atomic radius and reduced π-overlap of silicon compared to carbon. The bond angles (C1-Si-C2, C1-Si-C3, and C2-Si-C4) are considerably compressed, ranging from 90.37° to 99.47°, which departs markedly from the near-planar geometry of the carbon and boron sites. This strong angular reduction is characteristic of a transition from sp^2^ to a more sp^3^-like hybridization around silicon, leading to pronounced pyramidalization and local disruption of the π-network.

### 3.2 IR spectrum

Computational studies of the infrared (IR) spectrum are critical for molecular design, providing insight into a molecule’s vibrational distribution, bonding character, or structure before it is synthesized. Comparing the theoretically calculated IR spectrum with the experimental IR spectrum enables more accurate characterization of the designed structure (Amadei et al., 2010). Here, we calculated the IR spectra of C_60_ and its B- and Si-doped forms. The IR spectrum for C_60_ was then compared with its experimental spectrum to assess the accuracy of the computational method used (Figure 3).

**FIGURE 3:**
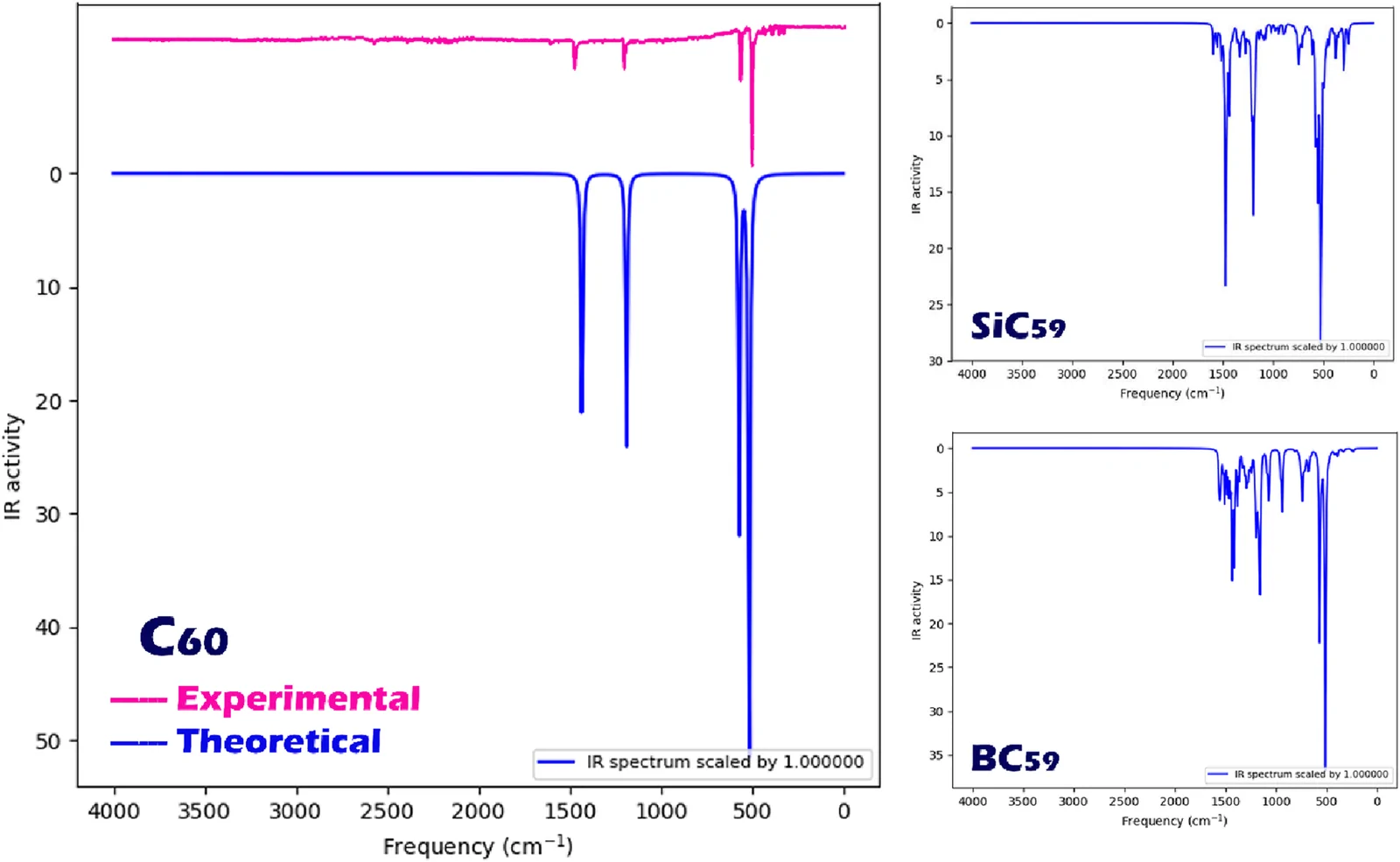
IR spectrum for C_60_ before/after doping with B (BC_59_) and Si (SiC_59_). Calculated using the B97D functional.

For pristine C_60_, the calculated spectrum reproduces the characteristic vibrational pattern expected for a highly symmetric icosahedral (Ih) molecule. Only a limited number of IR-active modes are observed due to symmetry restrictions. The theoretical spectrum displays the main absorption bands in the mid-IR region, particularly around ∼1,400–1,500 cm^−1^ and ∼500–600 cm^−1^, which correspond to the well-known T1u vibrational modes of C_60_ associated with C=C stretching and cage deformation vibrations.

Comparison with the experimental IR spectrum reported by Mauron et al. shows very good agreement in terms of both the number and positions of the main absorption bands (Mauron et al., 2012). Experimentally, pristine C_60_ exhibits four characteristic IR-active modes located approximately near 1,428, 1,183, 577, and 528 cm^−1^. The calculated spectrum reproduces these features with only small frequency deviations, which are typical of harmonic DFT calculations and arise from neglect of anharmonicity and solid-state effects. Importantly, the relative intensity pattern and the absence of additional spurious bands confirm that the computational method correctly captures the symmetry and vibrational behavior of the C_60_ cage. This agreement validates the chosen theoretical level for subsequent analysis of the modified systems.

For the substituted structures (SiC_59_ and BC_59_), the IR spectra exhibit a clear increase in the number of active bands compared to pristine C_60_. This behavior is expected because substitution breaks the high Ih symmetry of the fullerene cage, lowering it to a lower-symmetry point group and activating additional vibrational modes. In both SiC_59_ and BC_59_, new bands appear in the 1,000–1,600 cm^−1^ region, corresponding to perturbed C-C stretching modes and heteroatom–carbon vibrations. In the low-frequency region (<600 cm^−1^), noticeable shifts and changes in intensity are observed, reflecting cage distortion and mass effects introduced by B or Si substitution (Annen et al., 1998; Zhang et al., 2023).

Overall, the calculated IR spectrum of C_60_ shows strong agreement with the experimental spectrum, confirming the reliability of the computational approach. The systematic spectral changes observed for BC_59_ and SiC_59_ are consistent with symmetry breaking and structural perturbation effects. Therefore, the vibrational analysis supports both the structural models and the accuracy of the theoretical method used in this study. It should be noted that all IR spectra presented in this study are raw (unscaled) harmonic vibrational frequencies calculated directly from the DFT/B97D/6–31G(d) level of theory. Also, no scaling factor was applied to the reported frequencies.

### 3.3 MEP contours

The assessment of molecular electrostatic potential (MEP) presents a robust method for visualizing the spatial distribution of charge over molecular surfaces and provides vital information about potential sites of chemical interaction. Areas of negative electrostatic potential, often displayed in red on MEP maps, correspond to regions of significant electron density and are associated with either electronegative atoms or localized lone-pair density, providing favorable sites for electrophilic attack or hydrogen-bond donation. In contrast, positive regions shown in blue represent electron-deficient regions, which are typically found near and/or associated with electropositive regions and have a strong tendency to attract nucleophiles or electron-rich systems. The intermediate green regions of MEP maps represent areas of near-neutral electrostatic character and low reactivity (Hadi and Shamlouei, 2024). In this example, MEP maps were computed for the isolated fullerene structures C_60_, BC_59_, and SiC_59_, as well as the drug Php, to illustrate potential interaction sites for designing Surface@Php complexes (see Figure 4).

**FIGURE 4:**
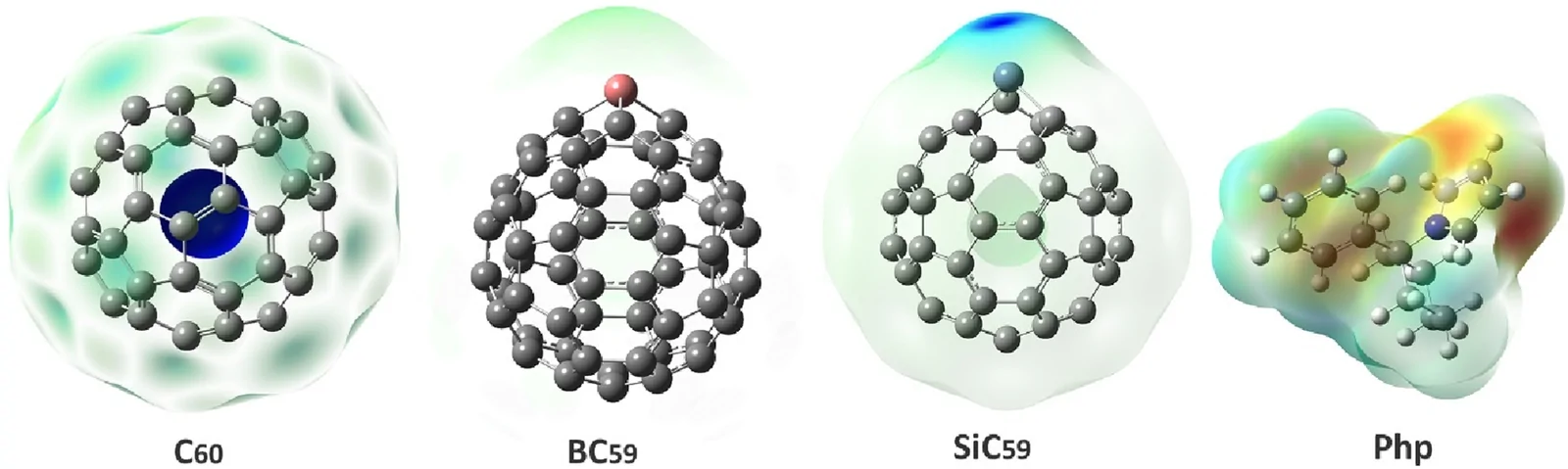
MEP maps of Php, pristine C_60_, BC_59_, and SiC_59_.

In C_60_, the overall distribution of blue color on the nanocavity surface indicates an electron-deficient characteristic throughout the molecule. In contrast, for BC_59_- and SiC59-doped fullerenes, the blue color is on the B and Si atoms, and the Si-doped site shows darker blue regions. This localized electron deficiency highlights the dopant atoms as the most electrophilic and reactive centers, making them the preferred sites for interaction with nucleophilic species.

For Php, the MEP map shows that the red color is localized on the nitrogen atom, indicating an electron-rich region that can act as a nucleophile. Combining these observations, the interaction between Php and the designed adsorbents is most likely to occur via the nitrogen atom of Php binding to the most electron-deficient region of the fullerene cages. In C_60_, this interaction may be distributed over the surface due to the uniform blue potential. However, the nitrogen atom of the Php is expected to interact preferentially with the boron or silicon impurity atoms (in BC_59_ and SiC_59_), as these regions exhibit the highest electron deficiency.

To avoid relying only on electrostatic-potential guidance for the adsorption geometry, several initial Php orientations were considered before selecting the final optimized complexes. For each adsorbent, Php was initially placed with different interaction modes, including the nitrogen atom directed toward the fullerene surface or dopant site, the phenyl ring oriented toward the fullerene cage to examine possible π-π/dispersion interactions, and the cyclohexyl/pyrrolidine side positioned toward the surface. All trial structures were subjected to geometry optimization under the same computational conditions, and the final complexes reported in Figure 5 correspond to the lowest-energy optimized geometries obtained from this orientation screening. Therefore, the MEP maps were used only as a qualitative guide for constructing plausible starting configurations, whereas the preferred adsorption geometries were selected based on optimized total energies and confirmed minimum-energy structures.

**FIGURE 5:**
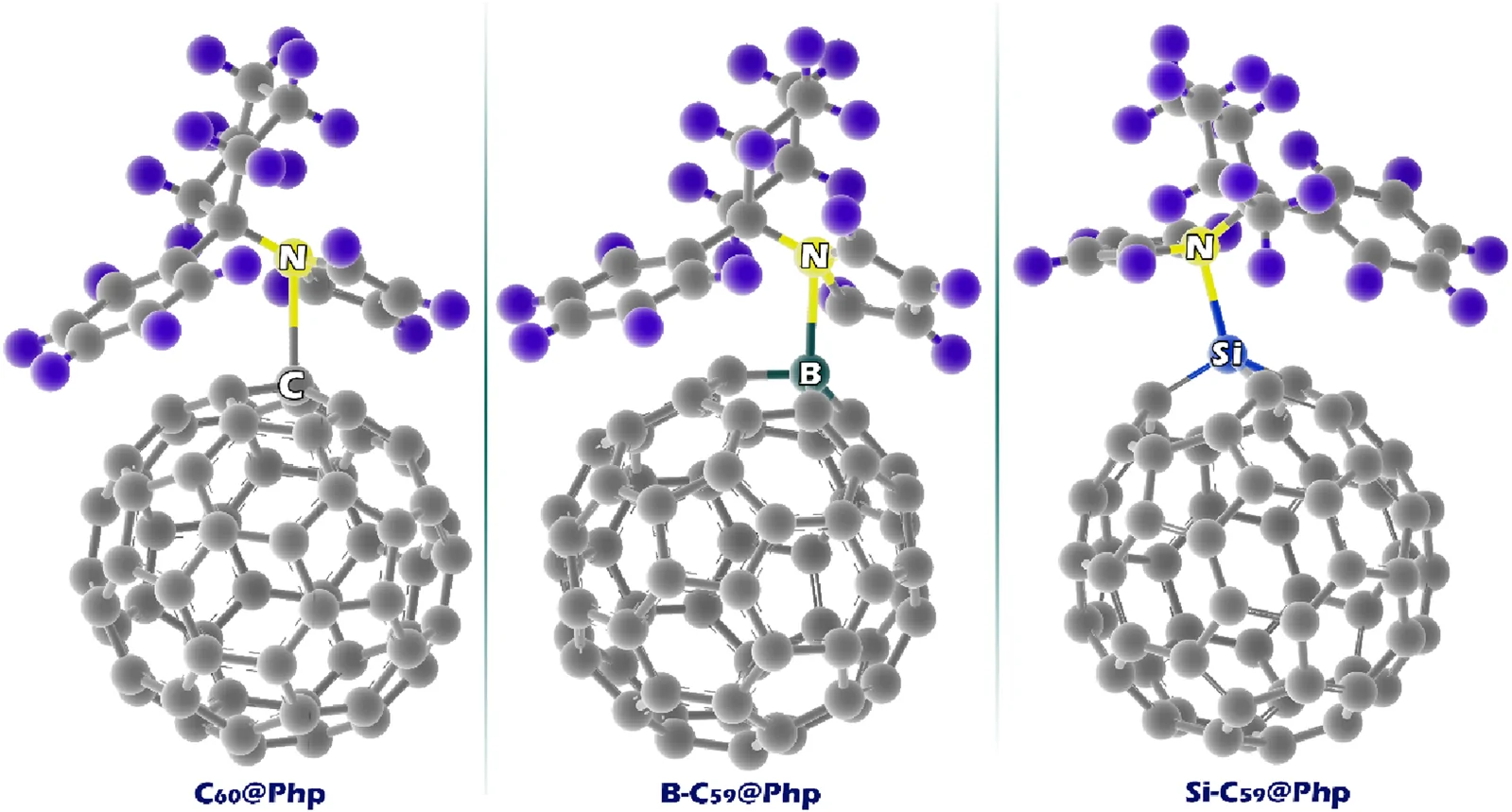
Optimized structure of each of the designed complexes.

### 3.4 Reactivity parameters

The HOMO–LUMO gap (HLG), chemical hardness (η), chemical softness (S), chemical potential (μ), maximum charge-acceptance descriptor (ΔNmax), and electrophilicity-based charge-transfer (ECT) descriptor were calculated to compare the electronic characteristics of the isolated structures and their Php complexes. These quantities are used here as comparative conceptual-DFT descriptors. They may indicate adsorption-induced electronic perturbation and charge-transfer tendencies but do not directly measure adsorption strength or practical sensing performance (Kebiroglu and Ak, 2025; Li et al., 2017; Pearson, 2005). The results are shown in Table 2.

**TABLE 2:** The values obtained for each of the reactivity parameters.

Structure	LUMO	HOMO	HLG	η	μ	S	∆N_max_	ECT
B97D/6-31G(d)								
C_60_	−3.58	−5.25	1.67 Theo	0.83	−4.41	0.59	5.28	------
			1.85 (Rabenau et al., 1993), 1.86 (Kremer et al., 1993), and 1.5 (Oshiyama et al., 1992) Exp					
BC_59_	−3.58	−4.93	1.35	0.67	−4.25	0.74	6.30	------
SiC_59_	−3.94	−5.12	1.18	0.59	−4.53	0.84	7.67	------
C_60_@Php	−3.49	−4.34	0.85	0.42	−3.91	1.17	9.21	−3.92
BC_59_@Php	−3._59_	−3.99	0.4	0.2	−3.79	2.5	18.95	−12.64
SiC_59_@Php	−3.23	−4.21	0.98	0.49	−3.72	1.02	7.59	0.086
B97D/6-311G(d,p)								
C_60_	−3.88	−5.54	1.66	0.83	−4.71	0.60	5.67	------
BC_59_	−3.88	−5.21	1.33	0.66	−4.54	0.75	6.87	------
SiC_59_	−4.19	−5.36	1.17	0.58	−4.77	0.85	8.22	------
C_60_@Php	−3.79	−4.58	0.79	0.39	−4.18	1.26	10.71	-2.86
BC_59_@Php	−3.87	−4.21	0.34	0.17	−4.04	2.94	23.76	-16.89
SiC_59_@Php	−3.54	−4.49	0.95	0.47	−4.01	1.05	8.53	-0.31
ωB97XD/6-31G(d)								
C_60_	−1.67	−7.72	6.05	3.02	−4.69	0.16	1.55	-----
BC_59_	−1.68	−7.4	5.72	2.86	−4.54	0.17	1.58	-----
SiC_59_	−2.06	−7.41	5.35	2.67	−4.73	0.18	1.77	-----
C_60_@Php	−1.65	−7.23	5.58	2.79	−4.44	0.17	1.59	-0.03
BC_59_@Php	−1.75	−6.53	4.78	2.39	−4.14	0.20	1.73	-0.14
SiC_59_@Php	−1.38	−6.42	5.04	2.52	−3.9	0.19	1.54	0.22

The calculated Kohn-Sham HOMO-LUMO gap of pristine C_60_ at the B97D/6–31G(d) level was 1.67 eV, which is numerically close to the previously reported experimental and theoretical values of approximately 1.5–1.86 eV (Figure 6). However, Kohn-Sham orbital-energy gaps are functional-dependent and are not strictly equivalent to experimentally measured optical or fundamental gaps. Therefore, this numerical agreement should be regarded as supportive evidence for the comparative analysis rather than as direct validation of the absolute B97D orbital gap.

**FIGURE 6:**
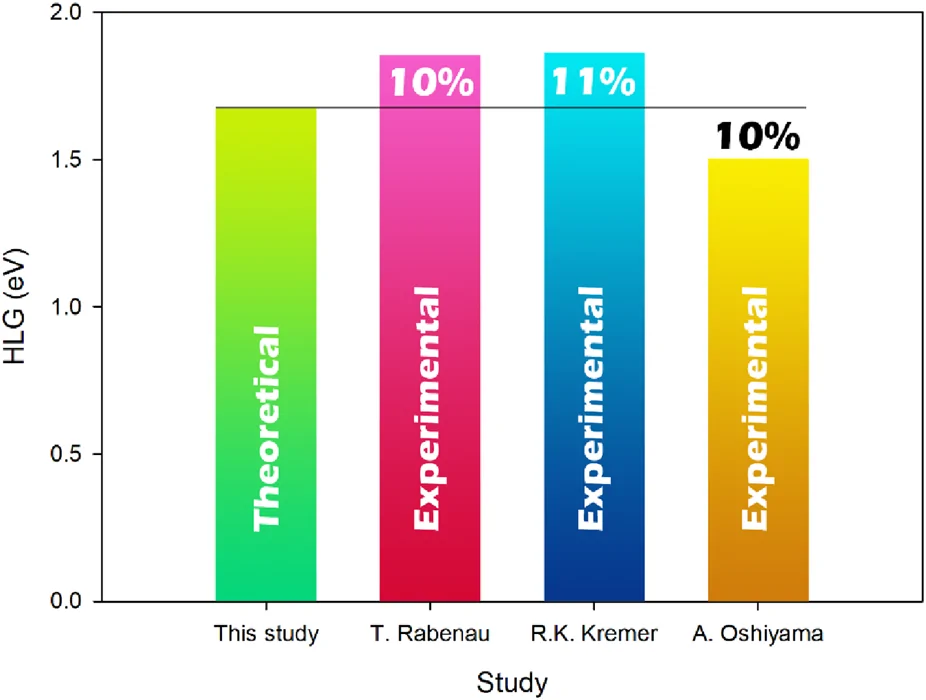
Comparison of the HLG value calculated with the B97D functional with experimental results from other literature.

As shown in Table 2, B and Si substitution reduce the HLG from 1.67 eV for C_60_ to 1.35 eV for BC_59_ and 1.18 eV for SiC_59_, respectively. After adsorption of Php, the HLG values decrease to 0.85, 0.40, and 0.98 eV for C_60_@Php, BC_59_@Php, and SiC_59_@Php, respectively. Among the complexes, BC59@Php shows the strongest electronic perturbation, with the smallest HLG, the highest softness and ΔNmax values, and a large negative ECT value, indicating pronounced charge transfer from Php toward the electron-deficient boron-doped fullerene.

The B97D/6-311G (d,p) and ωB97XD/6-31G(d) calculations reproduce the same qualitative trend, although the absolute HLG values differ because of the different basis-set and functional descriptions. In all cases, Php adsorption modifies the energy gap, and BC_59_@Php exhibits the largest calculated electronic perturbation among the studied complexes, making it a candidate for further experimental evaluation in sensing. Therefore, Figure 7 confirms that the electronic-response trend is not dependent on a single computational level.

**FIGURE 7:**
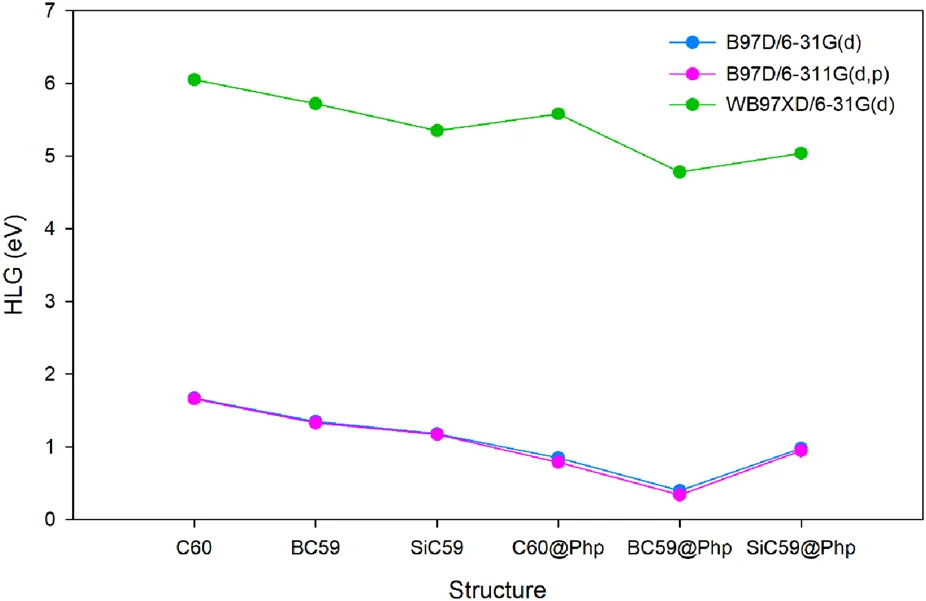
Comparison of the calculated HOMO–LUMO gaps obtained at the B97D/6-31G(d), B97D/6–311G (d,p), and ωB97XD/6-31G(d) levels. The relative adsorption-induced trends are generally preserved, although the absolute gaps are functional-dependent.

B97D is a dispersion-corrected semi-local GGA functional without Hartree-Fock exact exchange and is therefore more affected by self-interaction and electron-delocalization errors, which tend to compress the Kohn-Sham Frontier-orbital separation. In contrast, ωB97XD is a range-separated hybrid functional containing exact exchange, particularly at long range. This treatment improves the asymptotic exchange potential and generally stabilizes the occupied orbitals while shifting the virtual orbitals to higher energies relative to B97D, thereby producing systematically larger HOMO-LUMO gaps. For example, for pristine C_60_, the HOMO and LUMO energies change from −5.25 and −3.58 eV with B97D to −7.72 and −1.67 eV with ωB97XD, respectively, increasing the calculated gap from 1.67 to 6.05 eV. Similar offsets are observed for the other structures, giving HLG MAD and RMSD values of 4.35 eV. The nearly identical MAD and RMSD values indicate a largely systematic functional offset rather than random disagreement between the calculations. Consequently, the absolute Kohn-Sham gaps are functional-dependent and should not be considered directly interchangeable with experimental optical or fundamental gaps. The robust conclusion is instead the preservation of the relative ordering and adsorption-induced gap changes, with BC_59_@Php consistently exhibiting the strongest relative electronic response (Read references 23 and 28).

The calculated changes in the HOMO-LUMO gap, softness, and charge-transfer-related descriptors indicate that Php adsorption perturbs the electronic structures of the fullerene systems. BC_59_@Php exhibits the largest relative gap reduction among the studied complexes and may therefore be prioritized for subsequent experimental investigation of an electronic transduction response. Nevertheless, these descriptors alone do not establish sensitivity, selectivity, detection limit, or device performance, which depend on material preparation, electrode configuration, analyte concentration, matrix effects, kinetics, and operating conditions. DOS maps are reported as visual confirmation of the energy gap trends summarized in Supplementary Figure S2.

Based on Figure 8, the Projected Density of States (PDOS) analysis, together with the Total Density of States (TDOS), provides a comprehensive orbital-level description of the electronic structure across all systems. The PDOS plots deconvolute the TDOS into contributions from specific atomic orbitals (s, p, and d), offering a detailed view of how different atoms and orbital types participate in the Frontier molecular orbitals and influence the electronic properties upon doping and complexation. The PDOS contours are reported separately in Supplementary Figure S3.

**FIGURE 8:**
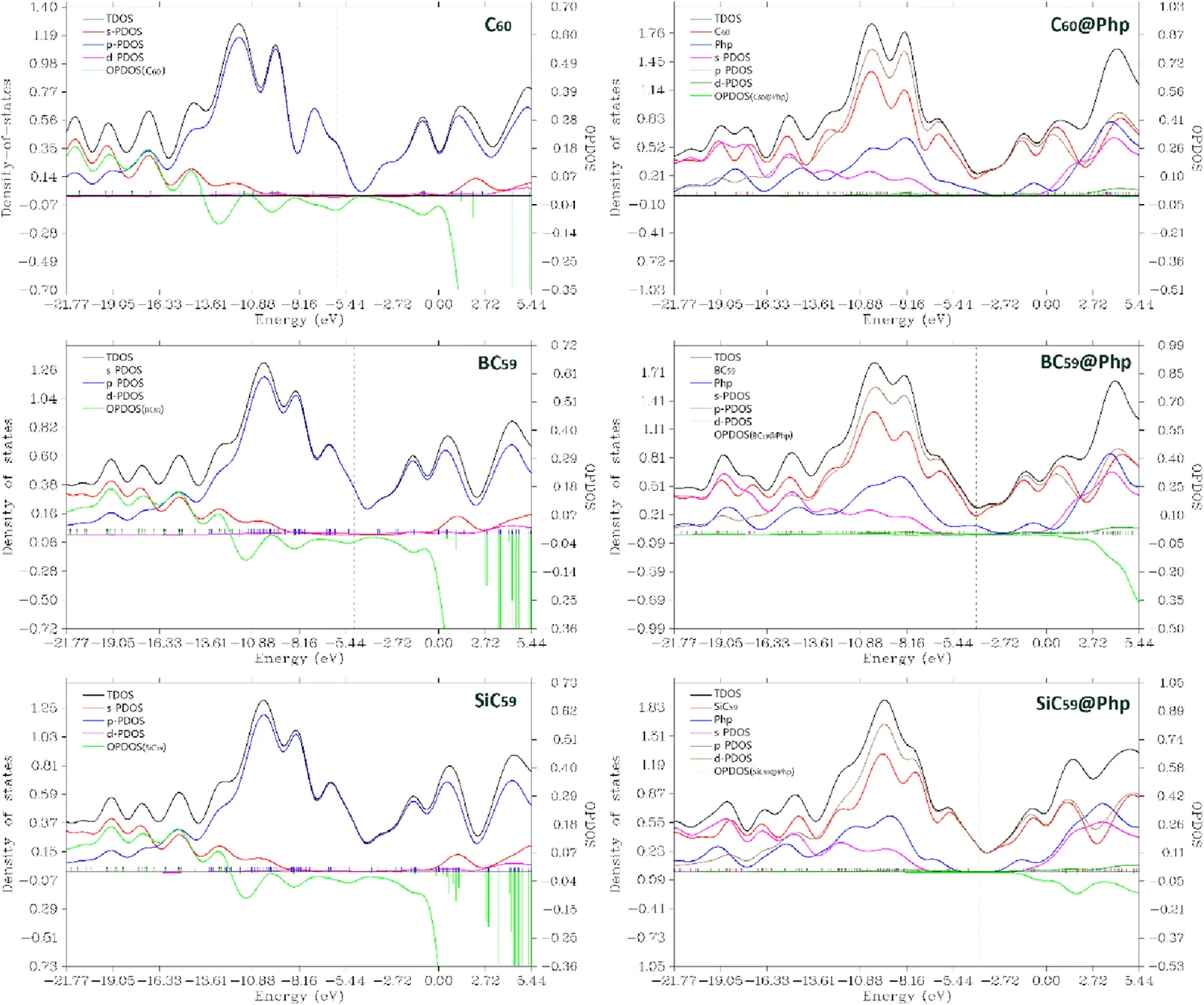
Total density of states (TDOS) plot and projected density of states (OPDOS) illustrating the individual contributions of s, p, and d orbitals in the Surface@Php complexes (Calculated with ωB97XD/6–31G(d)).

The TDOS/PDOS profiles show that the electronic structures of C_60_, BC_59_, and SiC_59_ are dominated by p-orbital states, whereas the s contribution is smaller and the d contribution is negligible. Substitutional doping modifies both the intensity and distribution of the fullerene states, particularly in the Frontier-energy region, confirming that B and Si atoms perturb the electronic structure of the carbon cage. After adsorption of Php, additional molecular states emerge and overlap with the fullerene-derived states across the occupied and low-lying unoccupied regions, producing clear changes in the TDOS line shapes. This redistribution is comparatively more pronounced for SiC_59_@Php, where the Php and SiC_59_ contributions exhibit broader and stronger overlap near the Frontier levels, together with noticeable OPDOS features. These observations suggest more effective orbital mixing and greater electronic communication between Php and the Si-doped surface than in _C60_@Php and BC_59_@Php. The OPDOS profile also indicates that the interaction contains both bonding and antibonding contributions, although the overall redistribution of states supports a comparatively more significant electronic interaction in SiC_59_@Php. However, these qualitative conclusions are interpreted more precisely together with the adsorption energy results in Section 3.7.

Evaluating the spatial distribution of the Frontier molecular orbitals provides insight into the regions of the molecule that will be most involved when electrons move during host-guest binding (i.e., electronic communication and charge migration). The contours of HOMO and LUMO distribution can be interpreted to readily show the electron-rich and electron-poor areas in the host, revealing the preferred sites of electronic donation (HOMO) and acceptance (LUMO). This mapping can improve understanding of the pathways of electronic communication and the structural domains that govern charge transfer and reactivity within the molecular assemblies investigated (Toriyama et al., 2022).

The distribution of the Frontier molecular orbitals (HOMO and LUMO) in the Php complexes provides insight into the electronic interactions and charge transfer behavior between the drug and the designed surfaces. The Frontier-orbital distributions provide a qualitative visualization of the regions involved in the adsorption-induced electronic response. In C60@Php, the Frontier orbitals remain comparatively delocalized over the molecular assembly; this delocalization should not be interpreted as evidence of structural instability. In BC59@Php, the orbital redistribution toward the boron-containing region is consistent with an electron-accepting contribution from the doped fullerene and a possible donor–acceptor pathway involving Php. In SiC59@Php, substantial fullerene-centered orbital localization and partial adsorbate contribution suggest pronounced electronic reorganization. These orbital patterns support the comparative charge-transfer interpretation but do not independently establish interaction strength or practical sensing capability (Figure 9).

- Quantitative Energy-Level Alignment and Charge-Transfer Analysis

**FIGURE 9:**
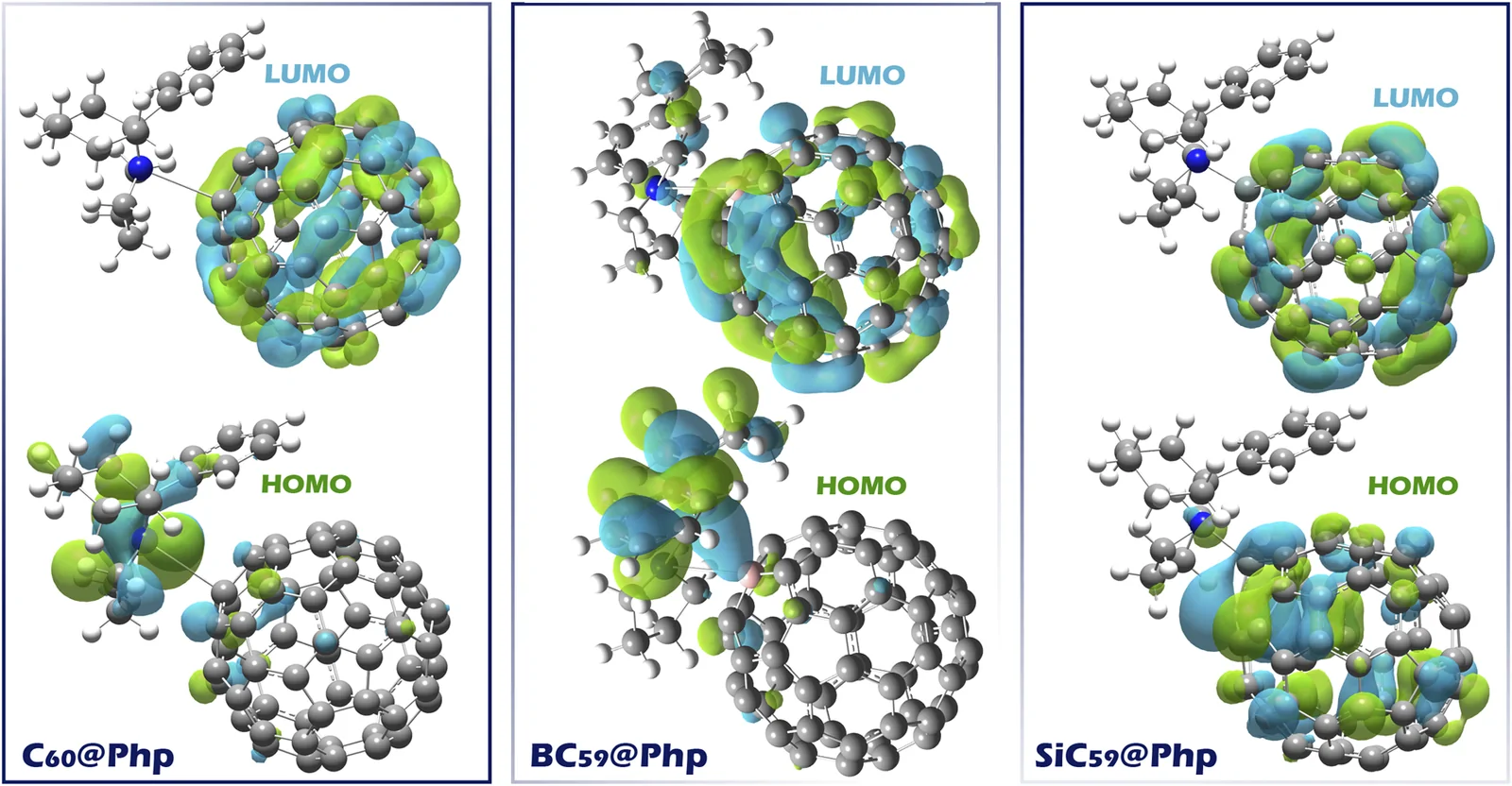
Distribution of Frontier molecular orbitals (HOMO and LUMO) in Php complexes.

To provide a quantitative interpretation of the DOS results, the Fermi level (EF) was approximated as the mid-gap energy using EF = (E_HOMO_ + E_LUMO_)/2. For pristine C_60_, EF is located at −4.41 eV. Upon boron and silicon substitution, EF shifts to −4.25 eV (BC_59_) and −4.53 eV (SiC_59_), respectively, indicating modification of the electronic density distribution induced by doping (see Supplementary Figure S2).

After complexation with Php, noticeable shifts in the Fermi level are observed. In BC_59_@Php, EF moves upward to −3.79 eV, whereas in SiC_59_@Php it shifts to −3.72 eV. This upward displacement reflects increased electron density near the conduction region and indicates a significant electronic perturbation due to analyte adsorption. These mid-gap-energy shifts provide a qualitative indication of adsorption-induced electronic perturbation; for the isolated molecular systems considered here, they should not be interpreted directly as experimentally measurable changes in carrier concentration.

Quantitatively, the reduction in the HOMO-LUMO gap further supports this behavior. The energy gap decreases from 1.35 eV to 0.40 eV for BC_59_ (≈70% reduction), from 1.18 eV to 0.98 eV for SiC_59_, and from 1.67 eV to 0.85 eV for pristine C_60_ upon adsorption. Since electrical conductivity is exponentially dependent on the band gap (σ ∝ e^(−Eg/2kT)^), the substantial narrowing of the band gap in BC59@Php yields the largest relative change in the gap-derived conductivity descriptor. This result indicates a potentially detectable electronic response but should not be interpreted as experimentally demonstrated sensing sensitivity.

The PDOS results quantitatively reveal that the newly formed states near EF in BC_59_ originate predominantly from boron 2p orbitals, while in SiC_59_ the LUMO region contains significant contributions from silicon 3p orbitals.

The PDOS profiles indicate adsorbate–surface orbital contributions near the Frontier region, including contributions from the dopant p states and the nitrogen-containing region of Php. These features are consistent with adsorption-induced orbital mixing but do not independently prove a specific donor-acceptor bond. The ECT descriptors suggest electron donation from Php toward BC59, as indicated by the negative ECT value of −12.64, whereas the small positive value calculated for SiC59@Php indicates a different and weaker charge-transfer tendency. These conceptual-DFT descriptors should therefore be interpreted qualitatively.

The quantitative Fermi-level shifts, gap modulation, PDOS orbital contributions, and charge-transfer descriptors collectively indicate that boron doping produces the largest electronic perturbation near EF and the greatest predicted relative variation in the gap-derived conductivity descriptor. This behavior identifies BC_59_ as a candidate for further sensing evaluation, although the magnitude and practical detectability of the response require experimental validation. In contrast, according to the reported results, silicon doping is predicted to result in stronger adsorption but a smaller relative gap-derived electronic response than boron doping, which explains the distinct sensing behaviors predicted for BC_59_ and SiC_59_. These results were further evaluated with the adsorption energy results in Section 3.7.

### 3.5 Dipole moment

The calculated dipole moments in Table 3 were examined as indicators of molecular polarity and adsorption-induced charge redistribution. An increase in dipole moment after complex formation suggests increased polarization of the molecular assembly; however, dipole-moment changes alone do not quantify charge transfer, adsorption strength, or practical sensing performance (Irfan et al., 2008; Liptay, 1974).

**TABLE 3:** Calculated values of dipole moments for (B/Si)-C_60_ and (B/Si)-C_60_@Php.

Structure	B97D/6–31G(d)	ωB97XD/6–31G(d)
C_60_	0.00	0.00
BC_59_	1.50	0.60
SiC_59_	0.35	0.62
C_60_@Php	4.86	1.68
BC_59_@Php	6.66	3.60
SiC_59_@Php	21.86	20.86

Pristine C_60_ exhibits a dipole moment of 0.00 Debye with both computational methods, which is fully consistent with its highly symmetric icosahedral structure and uniform charge distribution. Upon adsorption of Php, the dipole moment increases markedly to 4.86 Debye (B97D) and 1.68 Debye (ωB97XD). This substantial increase confirms that even the pristine fullerene undergoes measurable electronic polarization when interacting with the drug, indicating that C_60_ is inherently capable of engaging in electronic communication with Php, albeit to a moderate extent.

The isolated BC59 structure already displays a nonzero dipole moment of 1.50 Debye (B97D) and 0.60 Debye (ωB97XD), reflecting the asymmetry introduced by the heteroatom substitution. Upon complexation with Php, the dipole moment rises significantly to 6.66 Debye (B97D) and 3.60 Debye (ωB97XD). This enhancement is more pronounced than that observed for pristine C_60_, suggesting that boron doping facilitates stronger charge redistribution and electronic coupling between the sensor and the analyte. The boron atom, being electron-deficient, acts as an electrophilic center that attracts electron density from the nitrogen atom of Php, leading to enhanced polarization.

The most dramatic effect, however, is observed for the silicon-doped structure (SiC_59_). The isolated SiC_59_ has a modest dipole moment of 0.35 Debye (B97D) and 0.62 Debye (ωB97XD), indicating relatively low inherent polarity despite the presence of the dopant. Remarkably, upon adsorption of Php, the dipole moment increases to 21.86 Debye (B97D) and 20.86 Debye (ωB97XD). At the B97D/6-31G(d) level, this corresponds to an approximately 62-fold increase relative to isolated SiC59 and is substantially larger than the increases observed for C_60_ and BC_59_. Such a dramatic rise in dipole moment upon complexation signifies an exceptionally strong electronic perturbation and charge separation within the SiC_59_@Php complex. The silicon atom, with its larger atomic radius and more diffuse valence orbitals than boron and carbon, likely undergoes significant rehybridization upon interaction with Php, leading to pronounced charge transfer and polarization.

Figures 10B,C shows that the initial structures (C_60_, BC_59_, SiC_59_) have very small dipole moments, essentially close to zero. After Php adsorption, the calculated dipole moments increase for all three systems, with the largest increase observed for SiC59@Php, followed by BC59@Php and C60@Php. Both density functionals, B97D and ωB97XD, predict the same qualitative ordering (Figure 10A). The adsorption-induced dipole-moment increase follows the order SiC_59_@Php > BC_59_@Php > C_60_@Php using both density functionals. This trend suggests that SiC59@Php undergoes the largest calculated polarization and electronic reorganization. However, dipole moment alone is insufficient to rank adsorption strength or sensing performance; the interpretation must be combined with the BSSE-corrected adsorption energies, recovery-time estimates, and electronic-response descriptors.

**FIGURE 10:**
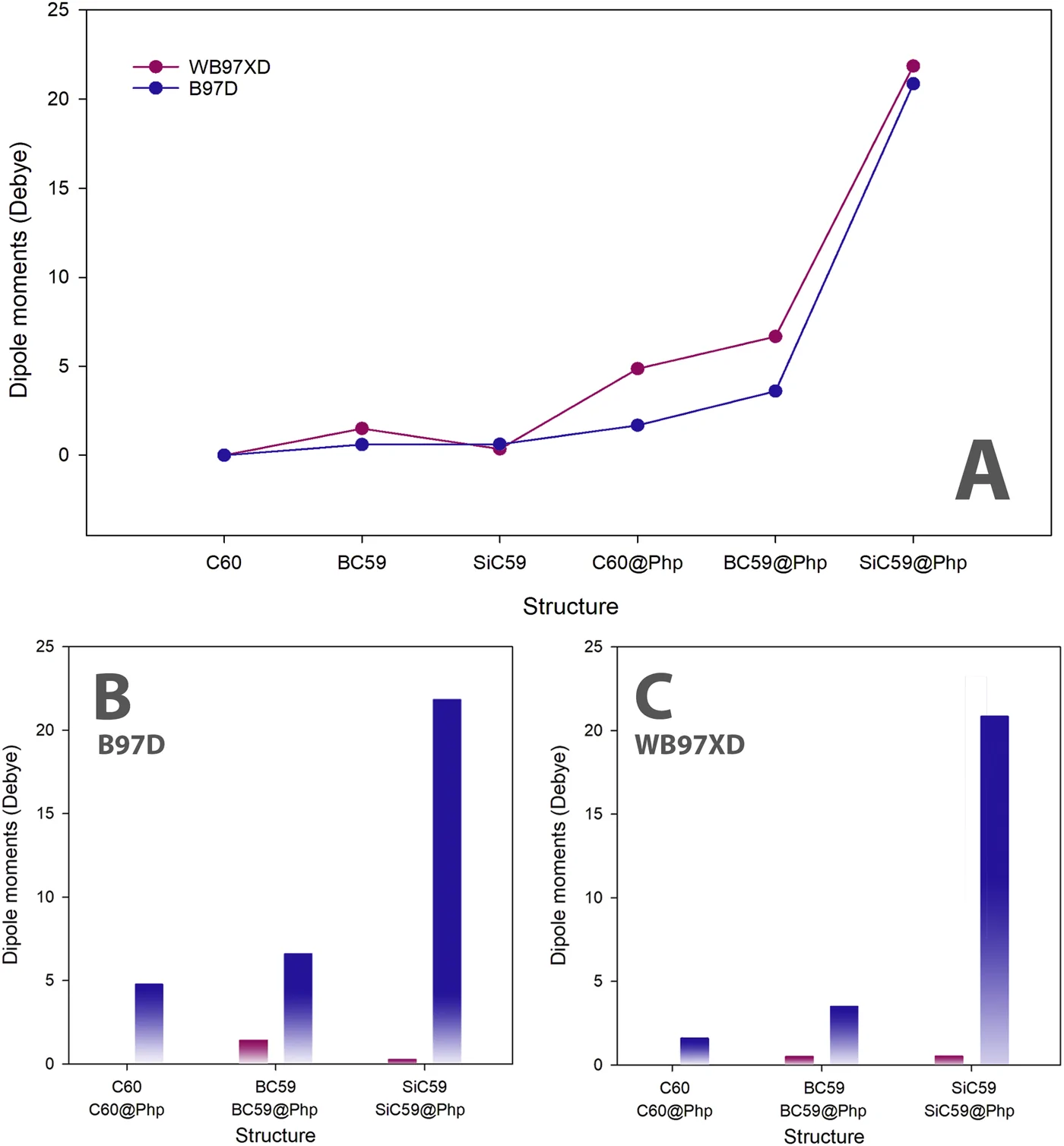
Comparison of the calculated dipole moments of C60, BC59, and SiC59 before and after Php adsorption: **(A)** comparison of the overall trends, **(B)** B97D/6-31G(d) results, and **(C)** ωB97XD/6-31G(d) results.

### 3.6 ESP analysis

Electrostatic-potential analysis (ESP) is useful for identifying electron-rich and electron-deficient regions that may participate in adsorbate-surface interactions. In ESP contours, colors represent the distribution of charge on a molecule’s surface. According to the color scale used in Figure 11, red represents regions of positive electrostatic potential, whereas blue represents regions of negative electrostatic potential. ESP analysis therefore provides qualitative guidance regarding the complementary electron-rich and electron-deficient regions that may participate in adsorbate–surface interactions. However, the maps alone do not quantify charge transfer or establish practical sensing performance (Ligorio et al., 2022; Ouafy et al., 2022).

**FIGURE 11:**
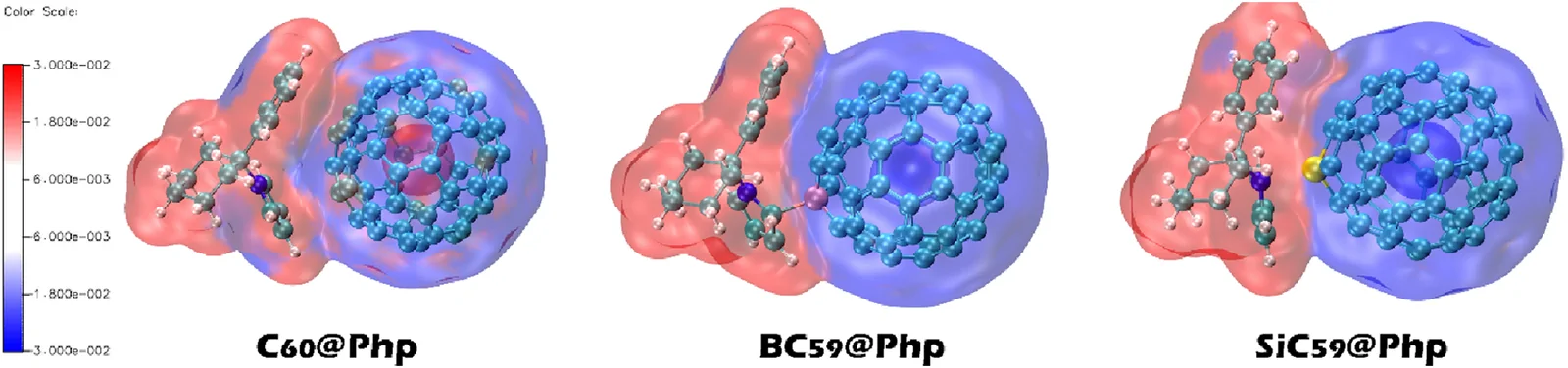
ESP contours for each of the complexes studied in this work.

The ESP maps in Figure 11 visualize the distribution of positive and negative electrostatic potential over the three optimized complexes. C60@Php exhibits a comparatively diffuse potential distribution, whereas the doped complexes show more localized potential contrast near the dopant-containing interaction regions. In BC_59_@Php, the complementary potential regions around the boron site and the nitrogen-containing part of Php are consistent with increased polarization and a localized donor–acceptor contribution. SiC_59_@Php exhibits the largest overall potential contrast, suggesting a substantial redistribution of electrostatic potential after complex formation.

The ESP trends are qualitatively consistent with the calculated dipole moments. At the B97D/6–31G(d) level, the dipole moments are 4.86, 6.66, and 21.86 D for C_60_@Php, BC_59_@Php, and SiC_59_@Php, respectively; the corresponding ωB97XD/6–31G(d) values are 1.68, 3.60, and 20.86 D. Thus, the Si-doped complex exhibits the largest calculated polarization using both functionals. Nevertheless, ESP maps and dipole moments provide qualitative indicators of charge redistribution and should not be treated as direct proof of charge-transfer magnitude, selectivity, or practical sensing capability.

### 3.7 Adsorption performance and sensing-related electronic response

BSSE-corrected adsorption energies (Eads), theoretical recovery-time (τ) estimates, and gap-derived conductivity descriptors (σ) were examined to compare adsorption strength, predicted reversibility, and adsorption-induced electronic perturbation. These quantities are used as comparative theoretical indicators and should not be interpreted as direct measurements of sensitivity, response time, reusability, or device-level electrical conductivity. Adsorption energy predicts the intensity of the interaction of molecules with the material surface, and recovery time predicts the time required for the analyte to leave the surface. Electrical conductivity also predicts the material’s ability to produce detectable electrical signals through molecular interactions. When combined, the three parameters provide a detailed evaluation of a material’s ability to serve as a sensing or adsorbing material (Zandi et al., 2026).

At the B97D/6–31G(d) level using CPCM water, the BSSE-corrected adsorption energies were −6.96, −8.04, and −38.79 kcal.mol^−1^ for C_60_@Php, BC_59_@Php, and SiC_59_@Php, respectively. Thus, relatively weak-to-moderate adsorption was predicted for C_60_@Php and BC_59_@Php, whereas SiC_59_@Php exhibited substantially stronger binding. The corresponding recovery-time estimates were 1.28 × 10^−7^, 7.97 × 10^−7^, and 2.81 × 10^16^ s, respectively. Accordingly, C_60_@Php and BC_59_@Php are predicted to undergo rapid desorption under the assumptions of the recovery-time model, while the extremely long recovery time of SiC_59_@Php indicates nearly irreversible adsorption. Therefore, SiC_59_ may be more suitable as a candidate material for strong Php adsorption or removal than as a reusable sensing platform (Table 4).

**TABLE 4:** BSSE-corrected adsorption energies, BSSE values, theoretical recovery-time estimates, and gap-derived conductivity descriptors of C_60_, BC_59_, SiC_59_, and their Php complexes. The recovery times reported in this table were calculated using the conventional fixed attempt-frequency prefactor ν0 = 10^12^ s^−1^. Results obtained using system-specific vibrational prefactors are reported separately in Supplementary Table S2.

Structure	E_ads_ (kcal.mol ^−^ ^1^)	BSSE (kcal.mol ^−^ ^1^)	τ (s)	Log_10_(τ)	σ (S/m)	Log_10_(σ)
B97D/6–31G(d)						
C_60_	-------	-------	-------	-------	2.33 × 10 ^−^ ^5^	−4.63
BC_59_	-------	-------	-------	-------	1.18 × 10 ^−^ ^2^	−1.92
SiC_59_	-------	-------	-------	-------	3.24 × 10 ^−^ ^1^	−0.48
C_60_@Php	-6.96	5.03	1.28 ×10 ^−^ ^7^	−6.88	2.00 × 10^2^	2.30
BC_59_@Php	-8.04	7.72	7.97 × 10 ^−^ ^7^	−6.09	1.27 × 10^6^	6.10
SiC_59_@Php	-38.79	6.36	2.81 × 10^16^	16.44	1.59 × 10^1^	1.20
ωB97XD/6-31G(d)						
C_60_	-------	-------	-------	-------	2.14 × 10 ^−^ ^42^	−41.66
BC_59_	-------	-------	-------	-------	1.32 × 10 ^−^ ^39^	−38.87
SiC_59_	-------	-------	-------	-------	1.77 × 10 ^−^ ^36^	−35.75
C_60_@Php	−1.41	3.48	1.08 × 10 ^−^ ^11^	−10.96	2.01 × 10 ^−^ ^38^	−37.69
BC_59_@Php	-3.52	6.92	3.87 × 10 ^−^ ^10^	-9.41	1.17 × 10 ^−^ ^31^	−30.93
SiC_59_@Php	-38.72	5.89	2.49 × 10^16^	16.39	7.43 × 10 ^−^ ^34^	−33.12
B97D/6-311G(d,p)						
C_60_	-------	-------	-------	-------	2.83 × 10 ^−^ ^5^	−4.54
BC_59_	-------	-------	-------	-------	1.74 × 10 ^−^ ^2^	−1.75
SiC_59_	-------	-------	-------	-------	3.94 × 10 ^−^ ^1^	−0.40
C_60_@Php	−7.78	3.38	5.11 × 10 ^−^ ^7^	−6.29	6.44 × 10^2^	2.80
BC_59_@Php	-11.86	4.35	5.04 × 10 ^−^ ^4^	-3.29	4.11 × 10^6^	6.61
SiC_59_@Php	-41.87	4.70	5.13 × 10^18^	18.71	2.85 × 10^1^	1.45
B97D/6-31G(d)/εr=76.2						
C_60_	-------	-------	-------	-------	2.33 × 10 ^−^ ^5^	−4.63
BC_59_	-------	-------	-------	-------	1.18 × 10 ^−^ ^2^	−1.92
SiC_59_	-------	-------	-------	-------	3.24 × 10 ^−^ ^1^	−0.48
C_60_@Php	−6.99	5.04	1.34 × 10 ^−^ ^7^	−6.87	2.00 × 10^2^	2.30
BC_59_@Php	−8.03	7.71	7.82 × 10 ^−^ ^7^	−6.10	1.27 × 10^6^	6.10
SiC_59_@Php	−38.84	6.37	3.07 × 10^16^	16.48	1.59 × 10^1^	1.20

The additional B97D/6–31G(d) single-point calculations using CPCM with εr = 76.2 produced adsorption energies of −6.99, −8.03, and −38.84 kcal.mol^−1^ for C_60_@Php, BC_59_@Php, and SiC_59_@Php, respectively. The absolute differences relative to the CPCM-water values were only 0.03, 0.01, and 0.05 kcal.mol^−1^. These negligible variations preserve the adsorption-strength order SiC_59_@Php ≫ BC_59_@Php > C_60_@Php and indicate that the principal adsorption conclusions are insensitive to this small change in the bulk dielectric constant. However, this comparison considers only continuum dielectric screening and does not represent the complete composition of blood or a physiological buffer.

Qualitatively, BC_59_@Php exhibits the largest calculated relative electronic change among the studied complexes, which may be favorable for producing a measurable transduction signal. Nevertheless, this theoretical trend does not establish experimental sensitivity, as the actual signal depends on electrode architecture, material loading, contact resistance, interfacial kinetics, analyte concentration, solvent and matrix composition, and measurement conditions.

The ωB97XD/6–31G(d) calculations reproduced the same qualitative adsorption-strength ordering, although the absolute values differed from those obtained using B97D. The BSSE-corrected adsorption energies were −1.41, −3.52, and −38.72 kcal.mol^−1^ for C_60_@Php, BC_59_@Php, and SiC_59_@Php, respectively. The corresponding recovery-time estimates were 1.08 × 10^−11^, 3.87 × 10^−10^, and 2.49 × 10^16^ s. Thus, both computational approaches predict rapid desorption for C60@Php and BC_59_@Php but exceptionally slow desorption for SiC_59_@Php. Although the absolute gap-derived conductivity values differ considerably between the functionals, BC_59_@Php exhibits the largest relative adsorption-induced conductivity change, supporting its selection as a candidate for further experimental sensing evaluation. The ωB97XD results preserve the same qualitative distinction, with rapid predicted desorption for C60@Php and BC59@Php and exceptionally slow predicted desorption for SiC59@Php. Again, the very long τ for SiC_59_@Php indicates strong and nearly irreversible adsorption, favoring removal and separation applications. The conductivity values, although much smaller in absolute magnitude than those predicted by B97D, display the same qualitative trend: BC_59_@Php exhibits the largest relative increase in σ upon adsorption, followed by SiC_59_@Php and C_60_@Php.

Both computational methods predict the same qualitative adsorption and structural trends despite differences in absolute values. SiC59@Php shows the strongest adsorption (Figure 12) and the longest recovery time (Figure 13A), supporting its potential use in removal and separation processes, as illustrated in Figures 14A–D. BC_59_@Php produces the largest relative electronic response (Figure 14A) while retaining moderate adsorption strength and acceptable predicted recovery under both attempt frequency (Supplementary Table S2), making it a promising candidate for experimental sensing evaluation. However, these theoretical results do not prove experimental sensitivity, which also depends on device architecture, interfaces, analyte concentration, matrix effects, and measurement conditions. Agreement between B97D and ωB97XD confirms the robustness of the relative trends, but not experimental sensing performance.

**FIGURE 12:**
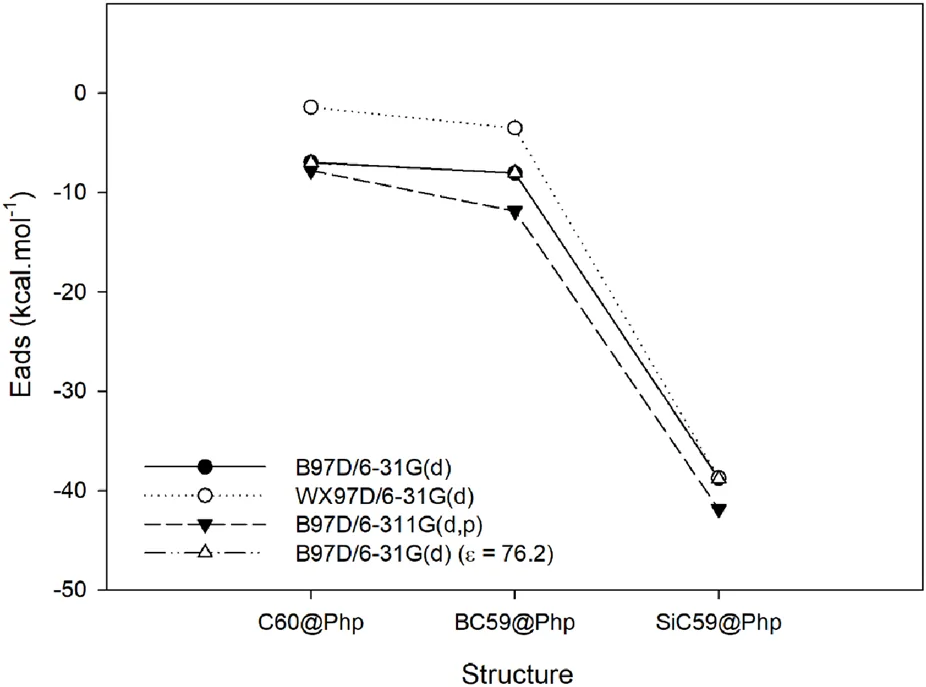
Comparison of the BSSE-corrected adsorption energies of Php on C_60_, BC_59_, and SiC_59_ calculated at the B97D/6–31G(d), ωB97XD/6–31G(d), and B97D/6–311G (d,p) levels using CPCM water, together with B97D/6–31G(d) CPCM calculations performed using εr = 76.2.

**FIGURE 13:**
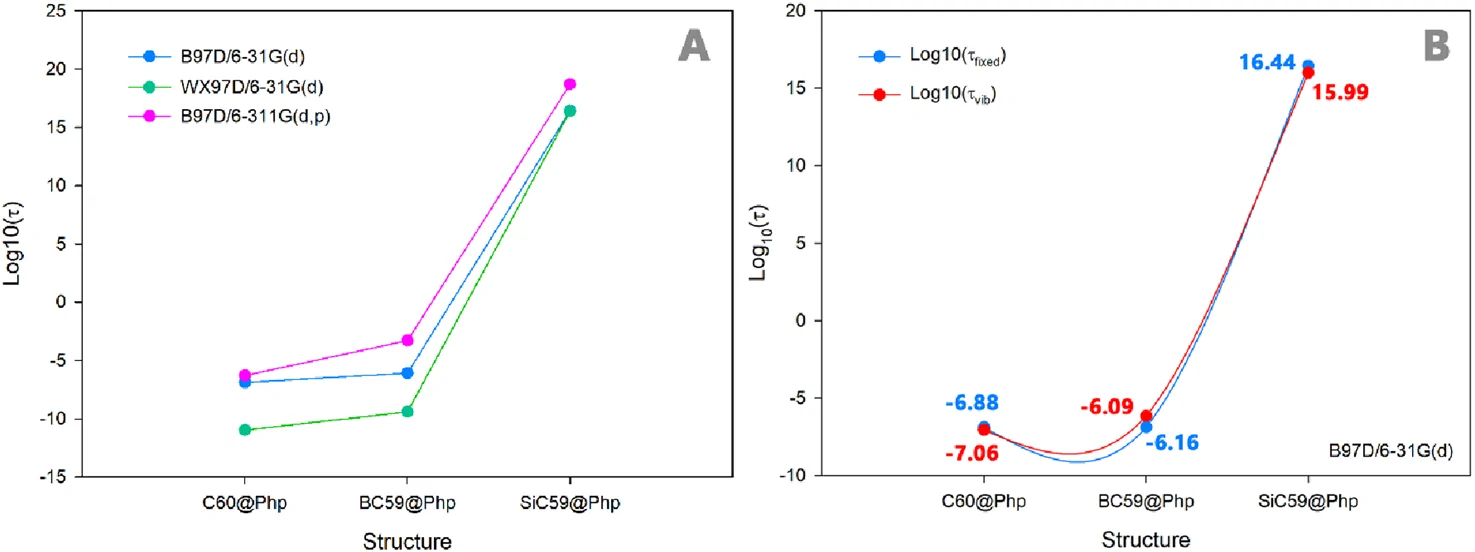
Comparison of log_10_(τ) for the Php complexes: **(A)** Recovery-time trends calculated at the B97D/6–31G(d), ωB97XD/6–31G(d), and B97D/6–311G (d,p) levels using the conventional fixed attempt-frequency prefactor. **(B)** Comparison of the B97D/6–31G(d) results obtained using the fixed prefactor ν_0_ = 10^12^ s^−1^ ( $\tau_{fixed}$ ) and the system-specific vibrationally derived prefactors ( $\tau_{vib}$ ).

**FIGURE 14:**
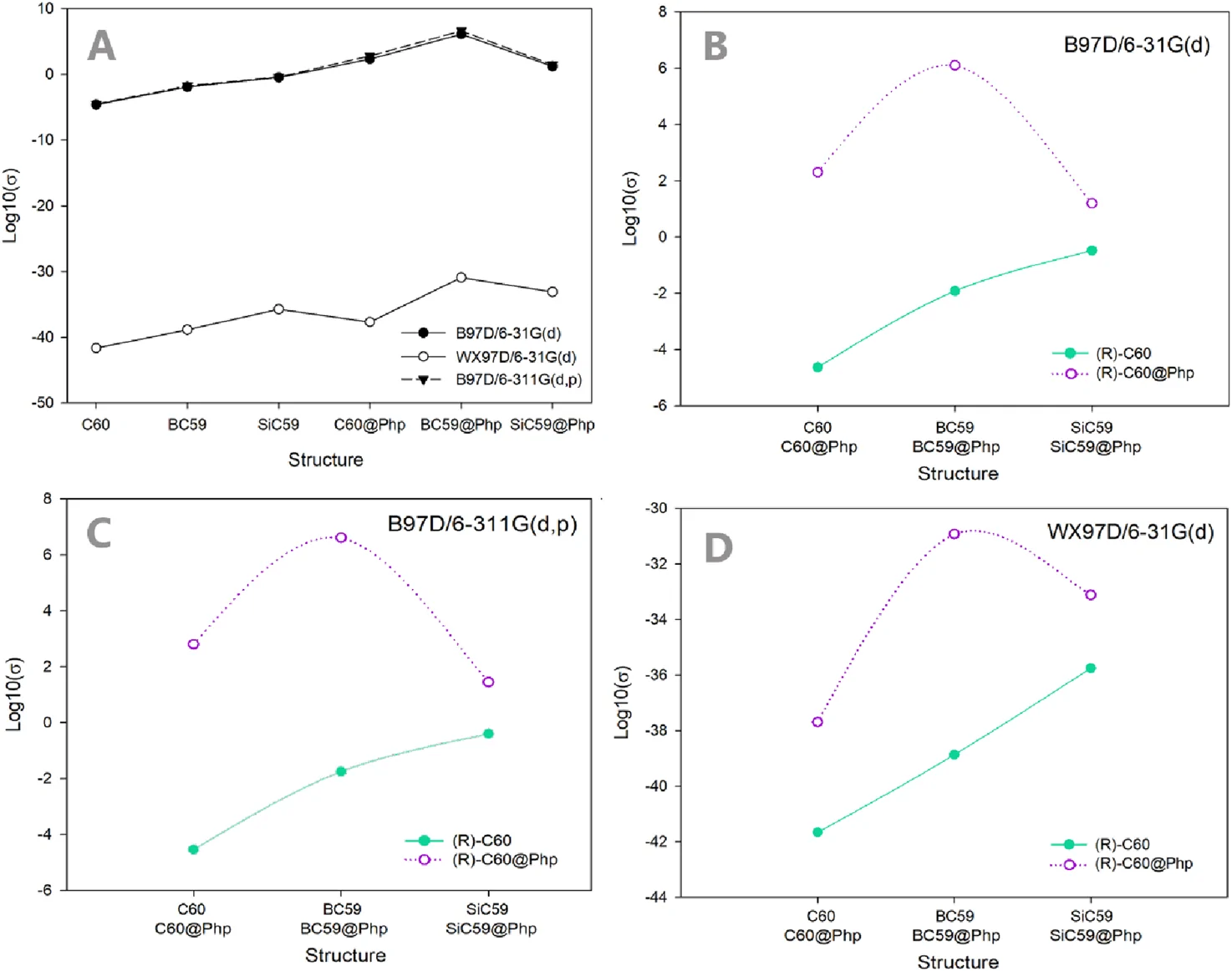
Comparison of the calculated electrical conductivity of pristine and doped fullerene structures before and after Php adsorption. **(A)** Logarithmic conductivity, log_10_(σ), of C_60_, BC_59_, SiC_59_, and their corresponding Php complexes, obtained using B97D/6–31G(d), ωB97XD/6–31G(d), and B97D/6–311G (d,p). **(B–D)** Corresponding conductivity-response profiles of the isolated fullerenes, R-C_60_, and Php-adsorbed systems, R-C_60_@Php, calculated at the B97D/6–31G(d), B97D/6–311G (d,p), and ωB97XD/6–31G(d) levels, respectively.

Also, Figure 13B compares the B97D/6–31G(d) recovery-time estimates obtained using the conventional fixed attempt frequency, ν0 = 10^12^ s^−1^, with those calculated using the system-specific vibrational attempt frequencies. The absolute difference between the log_10_(τ) value calculated using both attempt frequencies is negligible. The same recovery-time ordering, C_60_@Php < BC_59_@Php ≪ SiC_59_@Php, is retained by both treatments. Thus, use of the system-specific vibrational prefactors slightly changes the absolute recovery-time estimates but does not affect the qualitative conclusions. Both approaches predict rapid desorption for C_60_@Php and BC_59_@Php, whereas the very large positive log_10_(τ) obtained for SiC_59_@Php indicates exceptionally slow predicted desorption and strong Php retention (See Supplementary Table S2).

It is important to clarify that the electrical conductivity (σ) values reported in this work are not experimentally measured bulk conductivities and should not be interpreted as physically measurable transport properties of C_60_ or its doped derivatives. The σ values were estimated using a simplified thermally activated transport expression based on the HOMO-LUMO energy gap (Equation 9). Because this equation contains an exponential dependence on, E.g., small variations in the calculated gap can lead to numerically very large conductivity values. Such magnitudes (e.g., ∼10^9^ S.m^−1^) exceed realistic conductivity ranges for fullerene-based molecular materials and therefore represent only relative electronic descriptors rather than true charge-transport coefficients. The purpose of this calculation is to compare the relative change in electronic response before and after adsorption, not to predict absolute measurable conductivity. Consequently, discussion of σ in this work is strictly limited to qualitative trend analysis.

### 3.8 ELF and LOL

Electron Localization Function (ELF) and Localized Orbital Locator (LOL) maps were used to visualize adsorption-induced changes in electron localization across the investigated complexes. Regions of enhanced localization may identify areas of comparatively stronger electronic interaction, whereas weakly localized interfacial regions may be consistent with noncovalent contacts. However, ELF and LOL color patterns or numerical values alone cannot establish covalent bond formation, chemisorption, or a specific adsorption mechanism. Therefore, these maps are interpreted qualitatively together with the BSSE-corrected adsorption energies and NCI/RDG results (Figure 15).

**FIGURE 15:**
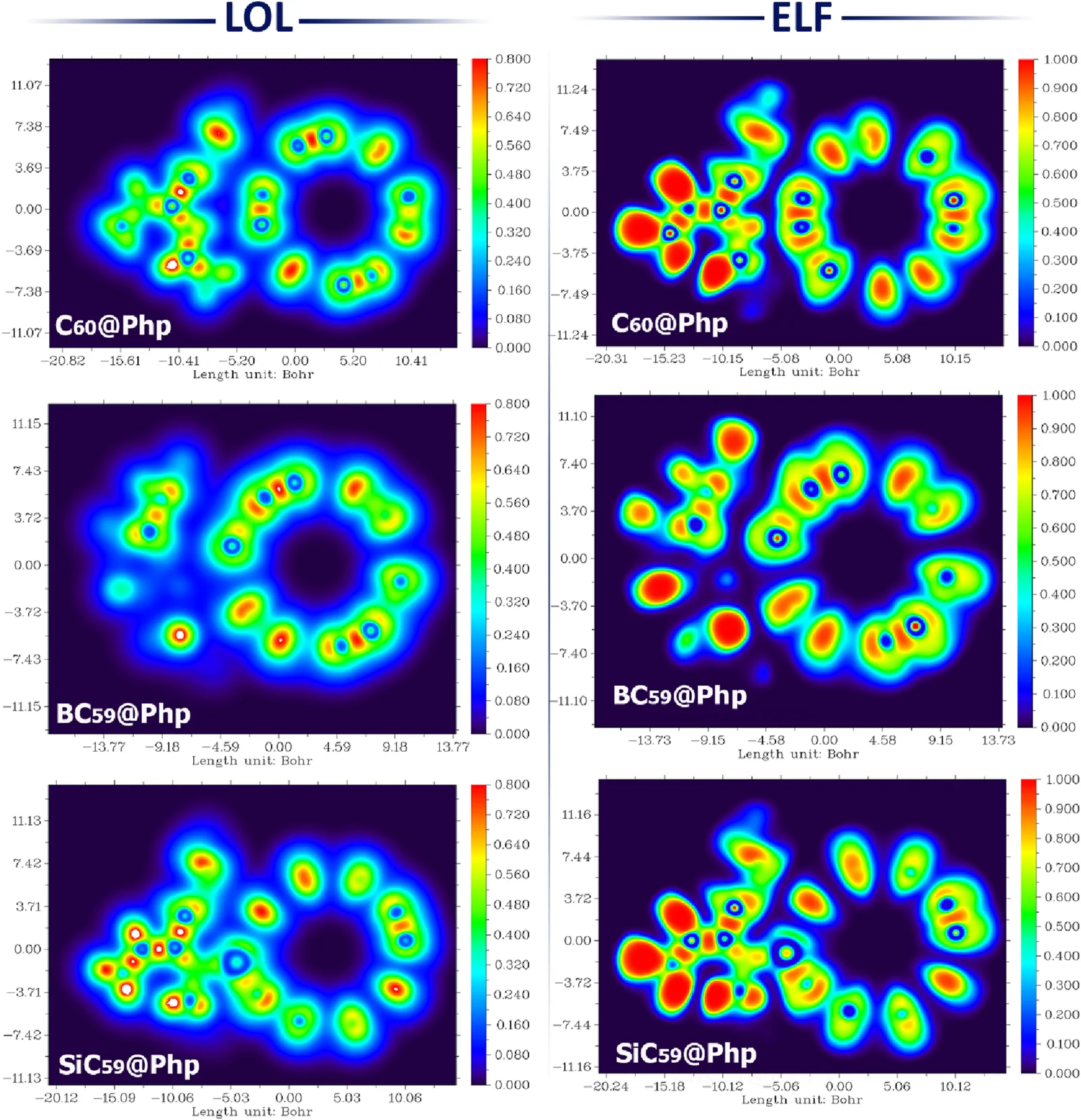
ELF and LOL maps calculated at the B97D/6–31G(d) level for C_60_@Php, BC_59_@Php, and SiC_59_@Php.

In pristine C_60_, the ELF and LOL maps exhibit a homogeneous electron distribution across the fullerene cage, consistent with its delocalized π-system and high symmetry. Upon complexation with Php, all systems show perturbations in electron localization around the interface, yet the extent and character of these changes vary markedly with dopant identity and align systematically with the adsorption energies.

For C_60_@Php, with a moderate BSSE-corrected adsorption energy of −6.96 kcal.mol^−1^ at the B97D/6–31G(d) level, the ELF and LOL contour maps predominantly green to light-blue regions at the contact zone, indicating weak electron localization typical of van der Waals and dispersive interactions. No significant charge concentration or depletion zones are observed, which is consistent with a predominantly noncovalent interaction. This is consistent with the NCI analysis, which identified green dispersive zones as the dominant stabilizing factor, and explains the relatively low adsorption strength and rapid recovery time.

In BC_59_@Php, which exhibits a higher BSSE-corrected adsorption energy of −8.04 kcal.mol^−1^ at the B97D/6–31G(d) level and the most negative ECT value (−12.64), the ELF and LOL maps reveal more pronounced changes. A distinct region of enhanced electron localization appears around the boron dopant site, accompanied by a complementary depletion zone near the nitrogen atom of Php. This pattern is consistent with partial charge redistribution and a localized donor-acceptor contribution, consistent with the strong blue NCI regions and the calculated dipole moment of 6.66 D at the B97D/6–31G(d) level. The localized ELF/LOL features indicate enhanced electron localization near the interaction region; however, the magnitude of ELF or LOL alone does not establish covalent or coordinative bond formation.

The most pronounced ELF and LOL changes are observed for SiC_59_@Php, which has the most negative BSSE-corrected adsorption energy of −38.79 kcal.mol^−1^ and the largest calculated dipole moment of 21.86 D at the B97D/6–31G(d) level. Here, the ELF map shows substantial electron redistribution, with intense localization zones around the silicon atom and along the Si-N interaction axis. LOL profiles similarly indicate significant orbital overlap and electron accumulation at the interface. These features suggest stronger localized electronic coupling than in the pristine complex. The elevated ELF values (>0.85) in the contact region, combined with the very long B97D/6–31G(d) recovery-time estimates of 2.81 × 10^16^ s using the fixed prefactor and 9.86 × 10^15^ s using the vibrationally derived prefactor and near-irreversible adsorption behavior, indicate that SiC_59_@Php engages in stronger electronic stabilization than purely physisorptive systems, explaining its exceptionally high adsorption energy.

The qualitative extent of electron redistribution at the fullerene-Php interface increases from C_60_@Php to the doped complexes, with the most pronounced changes observed for SiC_59_@Php. This trend is broadly consistent with the BSSE-corrected adsorption-energy ordering. However, ELF and LOL maps provide qualitative localization information and should not be used independently to assign bond type or practical sensing performance.

Taken together with the adsorption-energy and recovery-time results, the localization patterns support prioritizing SiC_59_ for further strong-adsorption or removal studies and BC_59_ for experimental evaluation as a potentially more reversible candidate sensing material.

### 3.9 NCI and RDG analysis

Both Noncovalent Interaction (NCI) and Reduced Density Gradient (RDG) diagrams are important tools for detecting, visualizing, and characterizing weak intermolecular interactions found in Surface@analyte complexes. These interactions include van der Waals Forces, hydrogen bonding, and steric repulsion. They make up a major part of the study of the mechanisms involved in adsorption or sensing. The reduced density gradient is a scalar function based on both electron density and its gradient; regions containing noncovalent interactions correspond to low values of RDG. In practice, RDG values are plotted against sign (λ_2_)ρ, where λ_2_ is the second eigenvalue of the Hessian matrix of the electron density. The plot distinguishes between different types of interactions: attractive interactions (e.g., hydrogen bonding/electrostatic attraction) are represented by negative values of sign (λ_2_)ρ; weak van der Waals interactions are represented by values of sign (λ_2_)ρ close to zero; and steric repulsions are represented by positive values of sign (λ_2_)ρ. NCI isosurfaces of these interactions are displayed in color. The blue surfaces represent strong attractive interactions; green surfaces represent weak van der Waals interactions; and red surfaces represent repulsive interactions. In adsorbent@analyte systems, the dominance of green isosurfaces typically confirms physisorption governed by dispersion forces, whereas the presence of blue regions suggests stronger interactions (Saleh et al., 2015). The NCI and RDG contours are shown in Figure 16.

**FIGURE 16:**
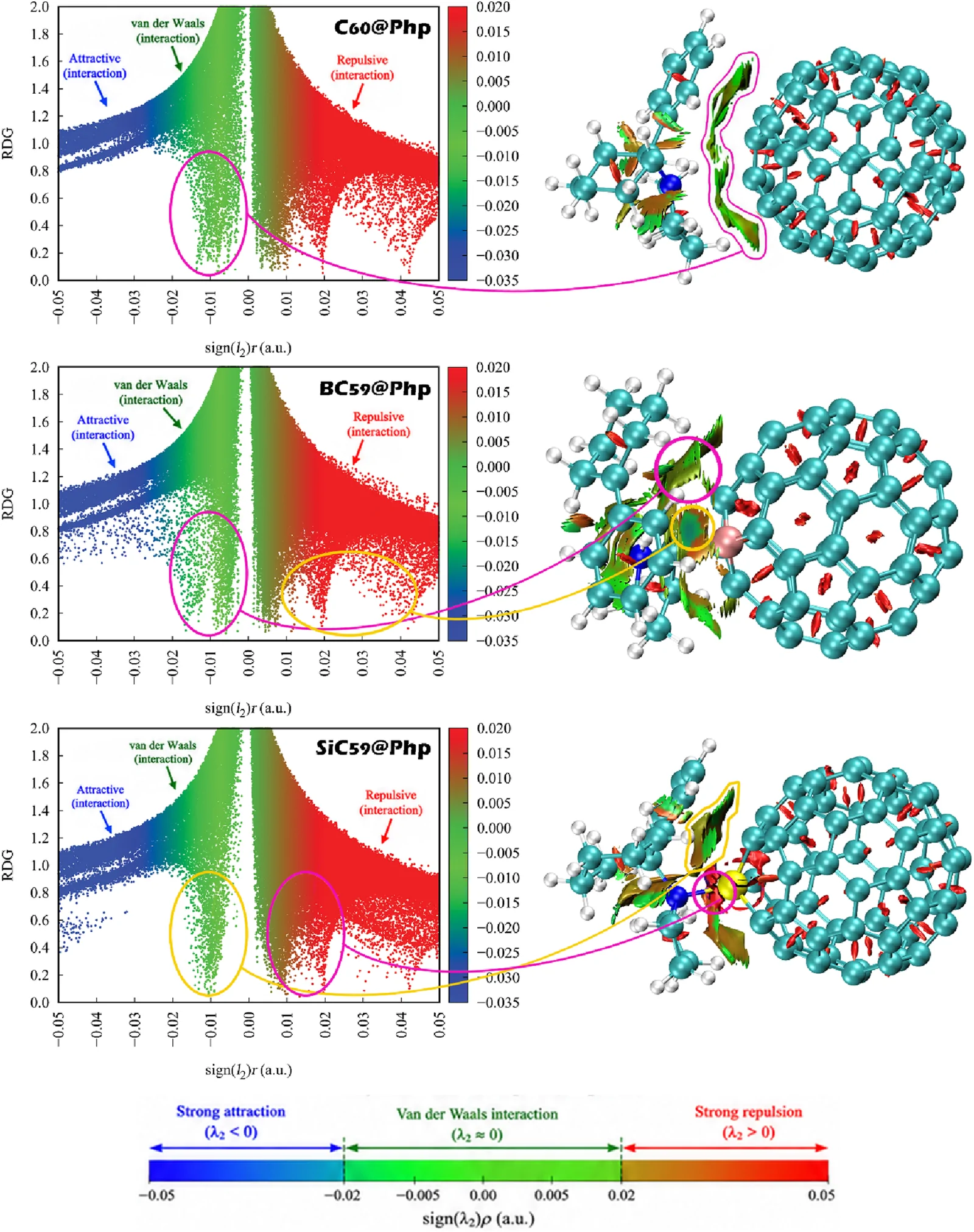
NCI/RDG plots and corresponding interaction isosurfaces for C_60_@Php, BC_59_@Php, and SiC_59_@Php.

The NCI/RDG results in Figure 16 provide a qualitative visualization of the weakly attractive, strongly attractive, and repulsive regions within the optimized complexes. These maps complement, but do not independently determine, the calculated adsorption energies.

For C_60_@Php, the BSSE-corrected adsorption energies are −6.96 kcal.mol^−1^ at B97D/6–31G(d) and −1.41 kcal.mol^−1^ at ωB97XD/6–31G(d). The interfacial region is dominated by green isosurfaces, which are consistent with dispersive and van der Waals contributions between Php and the delocalized fullerene surface. The smaller localized attractive regions suggest that electrostatic contributions are present but are not dominant.

For BC_59_@Php, the corrected adsorption energies are −8.04 and −3.52 kcal.mol^−1^ at the B97D and ωB97XD levels, respectively. A localized blue region is observed near the boron-containing interaction site and the nitrogen-containing region of Php. This feature is consistent with a localized attractive donor-acceptor or electrostatic contribution involving the electron-deficient boron site. The surrounding green regions indicate that dispersion also contributes to stabilization. Although the localized attractive feature is more pronounced than in C_60_@Php, BC59@Php does not exhibit the strongest overall corrected adsorption energy.

SiC_59_@Php exhibits the most negative corrected adsorption energies, −38.79 kcal.mol^−1^ at B97D/6–31G(d) and −38.72 kcal.mol^−1^ at ωB97XD/6–31G(d). Its NCI map contains both attractive blue regions near the Si-containing interaction site and extensive green dispersive regions over the contact area. The somewhat larger red regions indicate local steric compression associated with the closer contact geometry. These features are qualitatively consistent with a substantially stronger and more strongly polarized interaction than those predicted for C_60_@Php and BC_59_@Php.

Therefore, the BSSE-corrected adsorption-strength order obtained with both functionals is SiC_59_@Php ≫ BC_59_@Php > C_60_@Php. The NCI/RDG maps support distinct qualitative interaction patterns for the three complexes but should not be interpreted as experimental validation or unambiguous proof of a specific bond type.

### 3.10 Comparison with other literature

The findings of this study can be contextualized by comparison with recent computational investigations of doped C_60_ fullerenes for various applications in analyte detection and removal (Table 5). Mahdi et al. investigated the interaction between carboplatin and PEG-functionalized C_60_ as a drug delivery system, demonstrating that PEGylation significantly enhances binding stability and solubility in aqueous media, although quantitative adsorption energies were not reported (Mahdi et al., 2025). Alharby et al. examined p-xylene adsorption on B, N, and Si-doped C_60_ fullerenes for medical and environmental sensing, reporting adsorption energies of −4.56 kcal.mol^−1^ for pristine C_60_, −9.12 kcal.mol^−1^ for BC_59_, −9.33 kcal.mol^−1^ for NC_59_, and −18.96 kcal.mol^−1^ for SiC_59_, with corresponding HLG values of 1.63 eV (C_60_), 1.39 eV (BC_59_), 1.31 eV (NC_59_), and 1.22 eV (SiC_59_) (See reference 59). Alamri et al. studied acetone detection using B, N, and Si-doped C_60_ nanocages for heart failure diagnosis, obtaining adsorption energies of −20.88 kj. mol^−1^ (−4.99 kcal.mol^−1^) for C_60_@acetone, −109.28 kj. mol^−1^ (−26.12 kcal.mol^−1^) for BC_59_@acetone, and −137.17 kcal.mol^−1^ (−32.78 kcal.mol^−1^) for SiC_59_@acetone, with the SiC_59_@acetone complex exhibiting a fast recovery time of 1.50 × 10^−10^ s ideal for reusable sensing (See reference 60). Mohammadi et al. explored hexachlorobenzene (HCB) adsorption on Si- and Ge-doped C_60_, reporting adsorption energies of −15.94 kcal.mol^−1^ for C_60_@HCB, −30.05 kcal.mol^−1^ for C_59_Si@HCB, and −34.77 kcal.mol^−1^ for C_59_Ge@HCB, with the doped systems showing near-irreversible adsorption suitable for environmental remediation (See reference 61).

**TABLE 5:** Comparison of the results reported in this work with other literature.

Feature	Present Study (Php)	Study 1: (Mahdi et al., 2025)	Study 2 P- (Alharby et al., 2026)	Study 3: (Alamri and Alafnan, 2025)	Study 4: (Mohammadi et al., 2023)
Analyte	Rolicyclidine (Php)	Carboplatin	P-Xylene	Acetone	Hexachlorobenzene (HCB)
Application	Theoretical screening for Php adsorption and sensing-related electronic response	Drug Delivery	Medical/Environmental Sensing	Medical Diagnosis/Environmental Remediation	Environmental Adsorption/Removal
Dopants Studied	B, Si	PEG-functionalization	B, N, Si	B, N, Si	Si, Ge
Computational Method	B97D/6-31G(d), B97D/6-311G(d,p), and ωB97XD/6-31G(d)	DFT (B3LYP-D3)/6-31G(d) + MD	DFT (B97D)/6-31G(d)	DFT (B97D)/6-31G(d), QTAIM	DFT (B3LYP-D3, ωB97XD)/6-311+G(d,p)
Solvent Model	Water (CPCM)	Water (PCM)	Water (CPCM)	Water (CPCM)	Gas Phase
Adsorption Energy (Eads)	C60@Php: -6.96/−1.41 kcal.mol ^−^ ^1^ BC59@Php: −8.04/−3.52 kcal.mol ^−^ ^1^ SiC59@Php: −38.79/−38.72 kcal.mol ^−^ ^1^ Values correspond to B97D/6-31G(d)/ωB97XD/6-31G(d), with BSSE correction.	C_60_-Carboplatin: Not reported PEG-C_60_-Carboplatin: More stable than pristine (specific Eads not quantified)	C_60_@p-xylene: −4.56 kcal.mol ^−^ ^1^ BC_59_@p-xylene: −9.12 NC_59_@p-xylene: −9.33 SiC_59_@p-xylene: −18.96	C_60_@acetone: −20.88 kJ.mol ^−^ ^1^ (−4.99 kcal.mol ^−^ ^1^) BC_59_@acetone: −109.28 kJ.mol ^−^ ^1^ (−26.12 kcal.mol ^−^ ^1^) NC_59_@acetone:−24.62 kJ.mol ^−^ ^1^ (−5.88 kcal.mol ^−^ ^1^) SiC_59_@acetone: −137.17 kJ.mol ^−^ ^1^ (−32.78 kcal.mol ^−^ ^1^)	C_60_@HCB: −15.94 kcal.mol ^−^ ^1^ (B3LYP) C_59_Si@HCB: −30.05 C_59_Ge@HCB: −34.77
HOMO-LUMO Gap (HLG)	C_60_: 1.67 eV (B97D)/6.05 (ωB97XD) BC_59_: 1.35/5.72 SiC_59_: 1.18/5.35 C_60_@Php: 0.85/5.58 BC_59_@Php: 0.40/4.78 SiC_59_@Php: 0.98/5.04	C_60_: Not reported PEG-C_60_: Not reported (focus on interaction energies and dynamics)	C_60_: 1.63 eV BC_59_: 1.39 NC_59_: 1.31 SiC_59_: 1.22 BC_59_@p-xylene: 1.21 (↓13%) NC_59_@p-xylene: 0.96 (↓27%) SiC_59_@p-xylene: 1.22 (no change)	C_60_: 1.67 eV BC_59_: 1.54 NC_59_: 1.68 SiC_59_: 0.74 C_60_@acetone: 1.35 (↓19%) BC_59_@acetone: 1.66 (↑7.8%) NC_59_@acetone: 1.58 (↓6%) SiC_59_@acetone: 1._59_ (↑115%)	C_60_: ∼2.76 eV (B3LYP) C_59_Si: ∼2.50 C_59_Ge: ∼2.33 All complexes: Slight reductions upon HCB adsorption
Dipole Moment (μ)	C_60_: 0.00 D BC_59_: 1.50 SiC_59_: 0.35 C_60_@Php: 4.86 BC_59_@Php: 6.66 SiC_59_@Php: 21.86	Not reported	C_60_: 0.00 D BC_59_: 2.83 NC_59_: 2.67 SiC_59_: 2.16 BC_59_@p-xylene: 4.58 NC_59_@p-xylene: 5.81 SiC_59_@p-xylene: 7.51	C_60_: 0.00 D BC_59_: 0.51 NC_59_: 2.09 SiC_59_: 0.77 C_60_@acetone: 1.70 BC_59_@acetone: 4.42 NC_59_@acetone: 2.51 SiC_59_@acetone: 19.55	C_60_: 0.00 D C_59_Si: 1.34 C_59_Ge: 1.23 C_60_@HCB: 1.19 C_59_Si@HCB: 3.11 C_59_Ge@HCB: 3.68
Recovery Time (τ)	C60@Php: 1.28 × 10 ^−^ ^7^/1.08 × 10 ^−^ ^11^ s BC59@Php: 7.97 × 10 ^−^ ^7^/3.87 × 10 ^−^ ^10^ s SiC59@Php: 2.81 × 10^16^/2.49 × 10^16^ s Fixed prefactor ν_0_ = 10^12^ s ^−^ ^1^; B97D/ωB97XD.	Not applicable (drug delivery system)	C_60_@p-xylene: 1.51 × 10 ^−^ ^9^ s BC_59_@p-xylene: 3.56 × 10 ^−^ ^6^ NC_59_@p-xylene: 5.15 × 10 ^−^ ^6^ SiC_59_@p-xylene: 1.67 × 10^12^	C_60_@acetone: 1.30 × 10 ^−^ ^9^ s BC_59_@acetone: 7.90 × 10^6^ NC_59_@acetone: 1.50 × 10 ^−^ ^8^ SiC_59_@acetone: 1.50 × 10 ^−^ ^10^	Not reported (implied extremely long for doped systems)
Conductivity (σ)/HLG Change	BC_59_@Php: ∼70% HLG reduction; σ increases from 1.18 × 10 ^−^ ^2^ to 1.27 × 10^6^ S.m ^−^ ^1^ (B97D trend)	Not reported	BC_59_@p-xylene: σ increases by factor of ∼10^3^ NC_59_@p-xylene: σ increases by factor of ∼10^5^	SiC_59_@acetone: σ increases by factor of ∼10^7^ (relative to pristine SiC_59_)	Not reported (focus on adsorption strength)
Best Sensing Candidate	BC59; candidate for experimental sensing evaluation because of its large relative electronic perturbation and rapid predicted recovery	------	NC_59_ and BC_59_ (significant HLG reduction and conductivity modulation)	SiC_59_ (high sensitivity, fastest recovery)	------
Best Adsorption/Removal Candidate	SiC59; strongest BSSE-corrected adsorption and extremely slow predicted recover	PEG-C_60_ (enhanced stability for delivery)	SiC_59_ (highest Eads, but very long τ)	BC_59_ (highest Eads for removal)	C_59_Ge (highest Eads)
Key Electronic/Mechanistic Insight	BC59@Php exhibits a localized B···N attractive region and the largest relative gap-derived electronic response, whereas SiC59@Php exhibits the strongest corrected adsorption.	PEGylation improves solubility and binding strength, making C_60_ a viable carboplatin carrier.	NCI analysis shows that B and N doping create stronger attractive regions (blue/green) with p-xylene than with pristine C_60_.	Si doping creates strong adsorption with fast recovery due to favorable orbital interactions; B doping gives the highest Eads for removal.	Ge and Si doping significantly enhances HCB adsorption through stronger orbital overlap and charge transfer.

Heteroatom substitution generally increases the calculated interaction of C60 with the selected analytes, although the magnitude of the effect depends on the analyte, dopant, computational method, solvation treatment, and BSSE-correction procedure. In the present study, the B97D/6–31G(d) BSSE-corrected adsorption energies are −6.96, −8.04, and −38.79 kcal.mol^−1^ for C_60_@Php, BC_59_@Php, and SiC_59_@Php, respectively. The corrected SiC59@Php value is more negative than the reported values for SiC_59_@p-xylene, SiC59@acetone, and GeC_59_@HCB. However, these cross-study comparisons should be regarded as qualitative because the cited studies employ different functionals, basis sets, phases, and correction procedures.

BC_59_@Php exhibits the largest relative HOMO-LUMO-gap reduction in the present study, decreasing from 1.35 to 0.40 eV at the B97D/6–31G(d) level. Correspondingly, its gap-derived conductivity descriptor changes from 1.18 × 10^−2^ to 1.27 × 10^6^ S.m^−1^. These values indicate a large theoretical adsorption-induced electronic perturbation but should not be interpreted as experimentally measured conductivity or sensitivity.

The calculated dipole moment of SiC_59_ increases from 0.35 D to 21.86 D after Php adsorption at the B97D/6–31G(d) level, corresponding to an approximately 62-fold increase. The fixed-prefactor recovery-time estimates are 1.28 × 10^−7^, 7.97 × 10^−7^, and 2.81 × 10^16^ s for C_60_@Php, BC_59_@Php, and SiC_59_@Php, respectively. The corresponding B97D vibrationally derived values are 8.79 × 10^−8^, 6.98 × 10^−7^, and 9.86 × 10^15^ s. Both treatments predict rapid desorption for C_60_@Php and BC_59_@Php and extremely slow desorption for SiC_59_@Php.

Thus, the present calculations suggest an application-dependent distinction: BC_59_ may be prioritized for experimental sensing evaluation because of its comparatively large electronic perturbation and rapid predicted recovery, whereas SiC_59_ may be prioritized for strong adsorption or removal studies. These assignments remain theoretical and require experimental validation.

### 3.11 Future outlook

To facilitate the translation of these computational predictions into practical sensing devices, several experimental validation routes are proposed. First, boron- and silicon-doped C_60_ fullerenes can be synthesized via arc discharge or chemical vapor deposition, then purified and characterized by Raman spectroscopy, X-ray photoelectron spectroscopy (XPS), and high-resolution transmission electron microscopy (HR-TEM) to confirm doping and structural integrity. Second, modified electrodes can be fabricated by drop-casting or electrodepositing the synthesized materials onto glassy carbon, gold, or screen-printed electrodes. Electrochemical characterization using cyclic voltammetry (CV), differential pulse voltammetry (DPV), and electrochemical impedance spectroscopy (EIS) would enable quantitative measurement of changes in conductivity upon exposure to Php across a range of concentrations. Third, Spectro-electrochemical testing, which combines UV-Vis absorption spectroscopy with simultaneous electrochemical control, could monitor changes in optical properties during drug interactions, providing complementary insights into charge-transfer processes. Fourth, surface plasmon resonance (SPR) or quartz crystal microbalance (QCM) measurements could offer real-time, label-free detection of analyte binding. Finally, the selectivity of the candidate materials should be evaluated experimentally against structurally related compounds and other interferents commonly present in biological and forensic matrices. Such experimental studies would determine whether the calculated trends can be reproduced in fabricated materials and would help establish practical protocols for Php detection.

### 3.12 Limitations of the computational model

The results of the present study should be interpreted as comparative molecular-level predictions rather than direct evidence of practical sensing or adsorption performance. First, the aqueous environment was represented using the CPCM continuum solvation model. Although this approach accounts for the bulk dielectric polarization of water, it does not explicitly describe individual solvent molecules, analyte-water and fullerene-water hydrogen-bonding interactions, solvent reorganization, ion pairing, pH, ionic strength, or competition with other constituents of biological and forensic matrices. Consequently, the calculated adsorption energies, charge-transfer parameters, and optimized interaction geometries may differ quantitatively from those obtained in explicit aqueous environments.

Second, the calculations were primarily based on static optimized geometries and harmonic frequency analyses. Molecular dynamics simulations and free-energy sampling were not performed. Therefore, temperature-dependent conformational fluctuations, solvent-mediated rearrangements, adsorption and desorption pathways, kinetic barriers, and the statistical distribution of accessible binding configurations were not explicitly considered. Although several initial orientations of Php were examined, this procedure represents only limited orientation screening. It cannot guarantee exhaustive exploration of the potential energy surface or the identification of the global minimum-energy configuration.

Third, C_60_, BC_59_, and SiC_59_ were modeled as isolated, idealized molecular cages containing a single substitutional dopant. Experimentally prepared materials may contain fullerene aggregates, structural defects, different dopant concentrations and distributions, surface oxidation or functionalization, interactions with electrode substrates, and varying analyte surface coverage. These material-level factors may alter adsorption affinity, charge transfer, conductivity, recovery behavior, and selectivity relative to the isolated molecular models considered here. In addition, the conductivity and recovery-time values were derived from simplified theoretical relationships and should be regarded as qualitative comparative indicators rather than direct device-level observables.

Finally, the predicted adsorption and sensing responses for Php have not yet been experimentally validated. The comparison of selected properties of pristine C_60_ with literature data and the agreement between the B97D and ωB97XD calculations support the internal consistency of the computational trends but do not constitute experimental validation of the proposed BC_59_- or SiC_59_-based systems. Accordingly, the present work should be regarded as a pre-experimental screening study that identifies promising candidate materials for further investigation. Future studies should include explicit-solvent molecular dynamics and free-energy calculations, competitive adsorption in realistic sample matrices, models of fullerene films or supported electrodes, and experimental measurements of synthesis, adsorption, selectivity, and electrochemical response.

### 3.13 Statistical validation of results

Mean absolute deviation (MAD), root-mean-square deviation (RMSD), and Pearson correlation coefficient (R) were used to quantify pairwise agreement among the B97D/6–31G(d), B97D/6–311G (d,p), and ωB97XD/6–31G(d) datasets. MAD and RMSD measure differences in absolute numerical values, whereas R measures preservation of the relative trend across the matched structures. Consequently, a high R value may coexist with a large MAD or RMSD when two computational levels differ by a largely systematic offset. The statistics were calculated separately for the HOMO-LUMO gap (HLG; N = 6), BSSE-corrected adsorption energy (Eads; N = 3), log_10_(τ) (N = 3), and log_10_(*σ*) (N = 6) using Equations 10–12.

For a set of N corresponding values xi and yi obtained for the same descriptor and molecular structures at two selected computational levels, the mean absolute deviation (MAD) was calculated as:

$$MAD=\frac{1}{N}\sum_{i=1}^{N}\left|x_{i}-y_{i}\right|$$ (10)

This quantity measures the average magnitude of the absolute differences between the two datasets.

The root-mean-square deviation (RMSD) was calculated according to:

$$RMSD=\sqrt{\frac{1}{N}\sum_{i=1}^{N}{\left(x_{i}-y_{i}\right)}^{2}}$$ (11)

This quantity gives greater weight to larger deviations and is therefore sensitive to isolated numerical discrepancies.

The Pearson correlation coefficient (R), which evaluates the strength and direction of the linear relationship between the paired datasets, was obtained from:

$$R=\sqrt{\frac{\sum_{i=1}^{N}\left(x_{i}-\bar{x}\right)\left(y_{i}-\bar{y}\right)}{\sum_{i=1}^{N}{\left(x_{i}-\bar{x}\right)}^{2}\sum_{i=1}^{N}{\left(y_{i}-\bar{y}\right)}^{2}}}$$ (12)

Here, xi and yi denote corresponding values of the same physical descriptor for system i, and x̄ and ȳ are the means of the two datasets being compared. Three pairwise comparisons were evaluated: (i) B97D/6–31G(d) versus B97D/6–311G (d,p), which isolates the effect of basis-set expansion within the same functional; (ii) B97D/6–31G(d) versus ωB97XD/6–31G(d), which isolates the functional effect at a common basis set; and (iii) B97D/6–311G (d,p) versus ωB97XD/6–31G(d), which provides an additional cross-level comparison. In every case, only matched structures and identical descriptors were paired.

The first block of Table 6 shows that expanding the B97D basis set from 6–31G(d) to 6–311G (d,p) produces very small changes in the electronic descriptors and preserves the calculated rankings. For HLG, the MAD and RMSD are only 0.03 and 0.04 eV, respectively, with R = 1.000. Similarly, log_10_(σ) gives MAD = 0.27, RMSD = 0.32, and R = 1.000. The BSSE-corrected adsorption energies remain strongly correlated (R = 0.997), with MAD = 2.57 kcal.mol-1 and RMSD = 2.87 kcal.mol^−1^, while log_10_(τ) gives MAD = 1.89, RMSD = 2.11, and R = 0.997. The somewhat larger deviations in Eads and log_10_(τ) reflect the stronger adsorption energies predicted with the larger basis set, particularly for BC_59_@Php and SiC_59_@Php, and the exponential dependence of recovery time on adsorption energy. Nevertheless, the same qualitative ordering is retained: SiC_59_@Php exhibits the strongest adsorption, whereas BC59@Php shows the largest adsorption-induced electronic perturbation.

**TABLE 6:** Pairwise statistical comparison among B97D/6–31G(d), B97D/6–311G (d,p), and ωB97XD/6–31G(d) for the HOMO-LUMO gap (HLG), BSSE-corrected adsorption energy (Eads), log_10_(τ), and log_10_(σ). MAD and RMSD quantify absolute numerical differences, whereas Pearson R quantifies preservation of relative trends. R values are rounded to 3 decimal places.

Descriptor	N	MAD	RMSD	Pearson R
B97D/6-31G(d) vs B97D/6-311G(d,p): basis-set comparison				
HLG (eV)	6	0.03	0.04	1.000
Eads (kcal.mol ^−^ ^1^)	3	2.57	2.87	0.997
log_10_(τ)	3	1.89	2.11	0.997
log_10_(σ)	6	0.27	0.32	1.000
B97D/6-31G(d) vs ωB97XD/6-31G(d): functional comparison at a common basis set				
HLG (eV)	6	4.35	4.35	0.872
Eads (kcal.mol ^−^ ^1^)	3	3.38	4.13	1.000
log_10_(τ)	3	2.48	3.04	1.000
log_10_(σ)	6	36.77	36.81	0.872
B97D/6-311G(d,p) vs ωB97XD/6-31G(d): additional cross-level comparison				
HLG (eV)	6	4.38	4.39	0.860
Eads (kcal.mol ^−^ ^1^)	3	5.95	6.33	0.998
log_10_(τ)	3	4.37	4.64	0.998
log_10_(σ)	6	37.03	37.08	0.860

Comparisons involving ωB97XD also preserve the principal trends but show larger absolute deviations because B97D and ωB97XD use fundamentally different exchange-correlation treatments. For B97D/6–31G(d) versus ωB97XD/6–31G(d), the Pearson coefficients are 0.872 for HLG and log_10_(σ), and approximately 1.000 for Eads and log_10_(τ). The corresponding comparison using B97D/6–311G (d,p) gives R = 0.860 for HLG and log_10_(σ), R = 0.998 for Eads, and R = 0.998 for log_10_(τ). The HLG MAD/RMSD values remain large (approximately 4.35–4.39 eV), and the log_10_(σ) deviations are correspondingly amplified (approximately 36.8–37.1), because conductivity was estimated exponentially from the functional-dependent gap. In contrast, the near-perfect Eads and log_10_(τ) correlations confirm preservation of the adsorption-strength ranking. Because only three complexes contribute to Eads and log_10_(τ), these very high R values should be interpreted as evidence of trend consistency rather than as proof of numerical equivalence.

Figure 17 visually summarizes the statistical comparisons reported in Table 6. Panel (A) shows that expanding the basis set from 6–31G(d) to 6–311G (d,p) within the B97D framework produces only small MAD and RMSD values and near-perfect Pearson correlations, confirming that the calculated trends are largely insensitive to basis-set size. The larger numerical deviations in panels (B) and (C) arise mainly because B97D is a dispersion-corrected semilocal functional, whereas ωB97XD is a range-separated hybrid functional containing long-range exact exchange. This difference in exchange treatment systematically shifts the Frontier-orbital energies and therefore affects the absolute HLG and gap-derived conductivity values. Nevertheless, the high Pearson correlations, particularly between Eads and log_10_(τ), demonstrate that both functionals preserve the same qualitative ordering and yield the same principal conclusions despite differences in absolute magnitude. Thus, Figure 17 qualitatively supports Table 6 by distinguishing absolute numerical differences from the preservation of the principal comparative trends.

**FIGURE 17:**
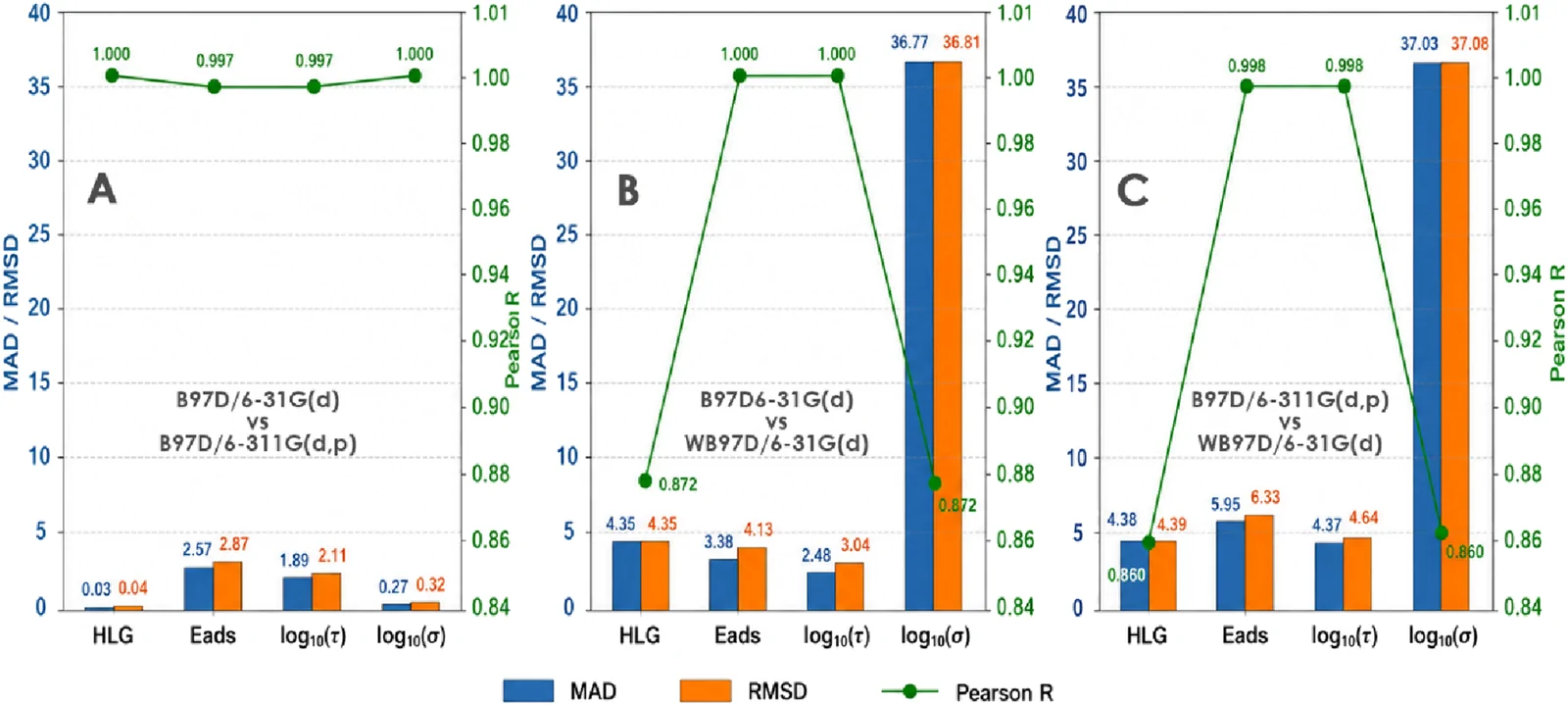
Pairwise statistical comparison of the HLG, BSSE-corrected adsorption energy (Eads), log_10_(τ), and log_10_(σ) obtained using **(A)** B97D/6–31G(d) and B97D/6–311G (d,p), **(B)** B97D/6–31G(d) and ωB97XD/6–31G(d), and **(C)** B97D/6–311G (d,p) and ωB97XD/6–31G(d). The blue and orange bars represent the mean absolute deviation (MAD) and root-mean-square deviation (RMSD), respectively, while the green line denotes the descriptor-specific Pearson correlation coefficient (R).

Overall, the three-level statistical validation supports the use of the present calculations as a comparative pre-experimental screening framework. The near-identity of the two B97D datasets demonstrates basis-set robustness, and the strong correlations with ωB97XD indicate that the principal structural rankings are preserved across different density-functional descriptions. However, MAD, RMSD, and R quantify only internal computational consistency; they do not constitute experimental validation or establish practical sensitivity, selectivity, detection limits, or device performance. The reported descriptors should therefore be used to prioritize candidate materials for subsequent synthesis and experimental evaluation.

## 4 Conclusion

This computational study compared pristine C_60_ and its substitutionally B- and Si-doped derivatives, BC_59_ and SiC_59_, as model materials for Php adsorption and sensing-related electronic response. Calculations performed at the B97D/6–31G(d), B97D/6–311G (d,p), and ωB97XD/6–31G(d) levels generally preserved the principal relative trends, although the absolute HOMO-LUMO gaps and weak adsorption energies remained method-dependent. These agreements therefore support internal computational consistency rather than experimental validation.

Structural analysis showed moderate local distortion around boron and substantially greater pyramidalization around silicon. At the B97D/6–31G(d) level, the HOMO-LUMO gaps of C_60_, BC_59_, and SiC_59_ were 1.67, 1.35, and 1.18 eV, respectively, and changed to 0.85, 0.40, and 0.98 eV after Php adsorption. BC_59_@Php consequently exhibited the largest relative gap reduction and the largest calculated change in the gap-derived conductivity descriptor, from log_10_(σ) = −1.92 to 6.10. These changes indicate a pronounced theoretical electronic perturbation but do not independently establish experimental sensitivity or conductivity.

The B97D/6–31G(d) BSSE-corrected adsorption energies were −6.96, −8.04, and −38.79 kcal.mol^−1^ for C_60_@Php, BC_59_@Php, and SiC_59_@Php, respectively. The same adsorption-strength order, SiC_59_@Php ≫ BC_59_@Php > C_60_@Php, was obtained at the B97D/6–311G (d,p) and ωB97XD/6–31G(d) levels. Using the conventional fixed attempt frequency, the corresponding B97D/6–31G(d) recovery-time estimates were 1.28 × 10^−7^, 7.97 × 10^−7^, and 2.81 × 10^16^ s. Also, the recovery time results calculated using the conventional constant-attempt frequency and the frequencies derived from the system’s specific vibrations showed negligible differences, confirming that the reported results are not sensitive to the selected prefactor.

The calculated dipole moments of the complexes were 4.86, 6.66, and 21.86 D at the B97D/6–31G(d) level, indicating the largest polarization for SiC_59_@Php. The ESP, ELF/LOL, and NCI/RDG analyses provide qualitative molecular-level insight into the interaction regions. C_60_@Php is dominated by dispersive contributions, BC_59_@Php contains a localized attractive region near the B···N contact, and SiC_59_@Php exhibits a combination of localized attractive and extensive dispersive contributions consistent with its substantially stronger adsorption. These topological features are not interpreted as independent proof of a specific type of bond or practical sensing performance.

Based on the combined theoretical results, BC_59_ may be prioritized for subsequent experimental evaluation as a potentially reversible candidate sensing material because it combines rapid predicted recovery with a comparatively large electronic response. SiC_59_ may be prioritized for strong adsorption or removal studies because of its substantially more negative corrected adsorption energy and extremely slow predicted desorption. The present work should therefore be regarded as a pre-synthesis computational screening study that narrows the candidate materials for future fabrication, selectivity testing, kinetic measurements, and electrochemical evaluation under realistic conditions.

## Data Availability

The original contributions presented in the study are included in the article/Supplementary Material, further inquiries can be directed to the corresponding author.
