# Supplementary material for: Theoretical insights into rolicyclidine adsorption and sensing using modified C60 fullerene
Source: Front Chem. 2026 Oct 5;14:1908245. doi: 10.3389/fchem.2026.1908245 (PMC13677873; doi:10.3389/fchem.2026.1908245)
Supplement: Supplementary file 1 [file DataSheet1.docx]

The XYZ coordinates of each of the designed structures were reported in Table S1.

Table S1. XYZ coordinates of the structures studied in this work.

C60

C	-0.07575757	-0.53977274	0.00000000

C	1.38813143	-0.53977274	0.00000000

C	2.08042043	0.65942526	0.00000000

C	1.34843843	1.92718326	-0.00001900

C	-0.03622957	1.92715126	0.00000500

C	-0.76813557	0.65935626	0.00002100

C	-0.52813757	-1.64615774	-0.84514300

C	0.65614843	-2.32990974	-1.36751300

C	1.84050843	-1.64611774	-0.84518700

C	2.96067943	-1.49341674	-1.64467700

C	3.26467543	0.82084826	-0.84525000

C	2.08032443	2.87210726	-0.84526500

C	1.38792143	3.76588526	-1.64470200

C	-0.07597157	3.76586226	-1.64464800

C	-0.76823757	2.87205826	-0.84515400

C	-1.95255957	2.18825126	-1.36745400

C	-1.95249757	0.82073426	-0.84510200

C	-2.38040357	-0.22576574	-1.64450700

C	-1.64840657	-1.49352474	-1.64452900

C	0.65609643	-2.82401474	-2.66101900

C	-0.52822957	-2.66264674	-3.50614400

C	-1.64844357	-2.01588874	-3.01203600

C	-2.38045757	-1.07098074	-3.85720300

C	-2.83282757	0.03537126	-3.01201000

C	-2.83287957	1.32888926	-3.50610800

C	-2.38051357	2.43526426	-2.66095700

C	-1.64862857	3.38022726	-3.50619100

C	-0.52840157	4.02703026	-3.01217300

C	3.26461443	2.18838226	-1.36763700

C	1.84017843	3.01056726	-5.67325800

C	2.96044843	2.85794526	-4.87388600

C	3.69242143	1.59019426	-4.87388000

C	3.26451943	0.54369926	-5.67330600

C	2.08018943	0.70507826	-6.51843700

C	-0.07609357	1.90418326	-6.51837200

C	-0.52846257	3.01054226	-5.67318700

C	0.65584643	3.69434326	-5.15088600

C	0.65588343	4.18844126	-3.85737800

C	1.84024243	4.02704626	-3.01221300

C	2.96046843	3.38030926	-3.50632700

C	4.14481643	1.32904426	-3.50637300

C	4.14487343	0.03555426	-3.01226300

C	3.69248043	-1.07084674	-3.85740200

C	3.26455243	-0.82382574	-5.15092200

C	2.08026843	-1.50762174	-5.67323000

C	1.34826643	-0.56272174	-6.51838300

C	-0.03640357	-0.56273774	-6.51832700

C	-0.76839057	0.70501226	-6.51833200

C	-1.64866357	2.85784926	-4.87370400

C	-2.38056257	1.59005626	-4.87365800

C	-1.95267757	0.54358026	-5.67310300

C	-1.95262257	-0.82394774	-5.15073900

C	-0.76829557	-1.50769074	-5.67312600

C	-0.07590757	-2.40146974	-4.87371500

C	1.38798543	-2.40144374	-4.87374700

C	1.84040043	-2.66257574	-3.50626500

C	2.96062143	-2.01577674	-3.01222400

C	3.69244343	2.43542626	-2.66118400

C	3.69255843	-0.22561574	-1.64469900

C	1.38781443	1.90422126	-6.51840400

C60@Php

C	1.29350263	-2.47575679	0.29863477

C	1.11969089	-1.96665207	1.69526610

C	-0.04950414	-2.27721487	2.39040415

C	-1.14526173	-3.15190227	1.78448699

C	-0.98535824	-3.60831168	0.50565452

C	0.27675380	-3.22757685	-0.26146549

C	2.17059031	-1.48299086	-0.48948001

C	1.87167790	-0.62566171	1.80466777

C	1.07814706	0.33622879	2.53812953

C	-0.71307892	-1.23087326	3.23535407

C	-2.50621275	-2.64517911	2.25940511

C	-3.60060143	-2.64876972	1.38520961

C	-3.41708351	-3.16430576	-0.05020899

C	-2.16115625	-3.61549114	-0.46091043

C	-1.60984345	-3.22596169	-1.83130719

C	-0.09895607	-2.98907904	-1.68755476

C	0.57620211	-2.00684606	-2.39751504

C	1.69596969	-1.26598764	-1.85666038

C	2.27595507	1.04301652	-0.12010685

C	1.81007102	1.25162632	-1.49909492

C	1.71570793	0.11934640	-2.55505216

C	0.35585290	0.15425964	-3.34367061

C	-0.25074237	-1.10900900	-3.33899241

C	-2.35466214	-2.43218417	-2.67261555

C	-3.73228693	-1.94260007	-2.24340379

C	-4.24614270	-2.28040144	-0.98845296

C	-2.24540413	-1.44403338	3.16840324

C	-4.83149572	1.30679205	0.29237168

C	-4.46436290	1.09852861	1.63432058

C	-3.35582439	1.96288271	2.26678434

C	-2.72425777	2.95060972	1.51461445

C	-3.12427653	3.18029865	0.04970584

C	-3.92750640	1.86202803	-1.96628712

C	-4.51500611	0.44769815	-2.02798282

C	-5.08025619	0.09814913	-0.63309795

C	-4.94866086	-1.20697518	-0.13650581

C	-4.54722310	-1.43528689	1.33664622

C	-4.31207394	-0.33632323	2.17769744

C	-2.51193147	1.06075500	3.18473939

C	-1.11674142	1.23594173	3.24786698

C	-0.45191422	2.30446535	2.43456564

C	-1.20223747	3.12011638	1.61172895

C	-0.67115939	3.44179616	0.23437870

C	-1.84030096	3.48929438	-0.73523994

C	-1.66235465	3.00269260	-2.01928297

C	-2.74707027	2.15262858	-2.66607412

C	-3.86693222	-0.53618364	-2.78480229

C	-2.55635688	-0.20638249	-3.53974535

C	-2.03796176	1.06677635	-3.48814810

C	-0.52200602	1.24913708	-3.36210785

C	-0.29357080	2.43499919	-2.44407035

C	0.77088120	2.40786750	-1.55096624

C	0.55152959	2.91626832	-0.17037971

C	1.40318784	2.04727548	0.72296324

C	0.89829981	1.74451815	1.97883374

C	-3.10146446	-0.34878441	3.13794943

C	-0.15266489	0.03463223	3.27157489

C	-4.12424440	2.39930351	-0.53569042

C	-1.66549962	-1.30703756	-3.46262979

C	4.55173847	4.34404827	0.94183242

C	4.70793814	2.97211180	0.71753535

C	4.81883636	2.08555791	1.79864810

C	4.74273980	2.57094306	3.11153688

C	4.60774771	3.95079360	3.33676633

C	4.51410161	4.83542061	2.25211091

H	4.45889458	5.01568413	0.11277050

H	4.75013581	2.59719924	-0.28352052

H	4.79505976	1.89566204	3.93880116

H	4.57518763	4.32922818	4.33751427

H	4.41853278	5.88696820	2.42407219

C	5.01704050	0.72259789	1.53026144

C	6.38037175	0.28153114	2.07834990

C	3.95090768	0.35261046	2.55881630

C	6.37861969	-1.25798048	2.12825519

H	7.16523727	0.64211693	1.44665929

C	3.87983467	-1.17794508	2.62213961

H	4.21327874	0.74313212	3.51986158

C	5.25274459	-1.72343217	3.09030774

H	3.64812040	-1.56319719	1.65073807

H	5.22121032	-2.79258843	3.11417747

H	6.52185594	0.66788742	3.06817124

H	6.20541336	-1.64650205	1.14718876

H	5.45627818	-1.34749937	4.07067273

H	3.12187540	-1.47892176	3.31392735

H	3.00617996	0.75824079	2.26083697

N	4.56244262	-0.45435280	0.76750008

C	5.28461395	-0.57612816	-0.51603749

C	4.81347439	-1.75507973	1.43192061

C	4.87140615	-1.99423504	-0.91700304

H	6.34571156	-0.53089870	-0.37049473

C	5.30265404	-2.73813721	0.35111820

H	4.67885083	-1.97129405	2.47273681

H	5.33405699	-2.36684978	-1.80639002

H	4.89847474	-3.72773645	0.43092939

H	7.32307708	-1.61350849	2.48865327

C	3.01047839	-0.36060637	0.51609511

H	5.01347228	0.17996393	-1.22465747

H	3.81032702	-2.03776313	-1.05609551

H	6.36881695	-2.80943638	0.39233095

H	5.16598007	1.00421204	0.50723528

BC59

C	-0.11472745	-0.55095891	0.09245325

C	1.42615096	-0.54776435	0.08854555

C	2.10521172	0.62738778	0.09233992

C	1.33817523	1.96636797	0.10790102

C	-0.03789419	1.96023747	0.12263805

C	-0.79695429	0.61914754	0.10412670

C	-0.58788866	-1.71331409	-0.80007172

C	0.65732904	-2.43107961	-1.35059994

C	1.90190237	-1.71082586	-0.80196511

C	2.99740023	-1.56207210	-1.58364969

C	3.35268362	0.79568898	-0.79917502

C	2.13275050	2.97084837	-0.78380909

C	1.47436799	3.86147527	-1.56014785

C	-0.83356169	2.95362485	-0.73804753

C	-2.06070239	2.23585664	-1.32081606

C	-2.04800187	0.78961006	-0.78860743

C	-2.45668231	-0.23185408	-1.57874055

C	-1.68400598	-1.56396160	-1.58224934

C	0.65770364	-2.91564142	-2.61555344

C	-0.58753008	-2.74597102	-3.50413896

C	-1.68264463	-2.11224478	-3.02037993

C	-2.45137066	-1.11766721	-3.90841388

C	-2.92873686	0.04561429	-3.01905553

C	-2.93256105	1.31381328	-3.50256046

C	-2.47117599	2.48541411	-2.61041081

C	-1.70460757	3.49371009	-3.52210643

C	-0.61702333	4.14643957	-3.05254228

C	3.36269851	2.23671031	-1.34270162

C	1.90312874	3.06924511	-5.72599279

C	3.00068244	2.91969745	-4.94279040

C	3.76758743	1.58379211	-4.94196054

C	3.34885287	0.55970067	-5.72358467

C	2.10393827	0.72932746	-6.61194244

C	-0.11232297	1.90320542	-6.61358568

C	-0.59072253	3.07059350	-5.73135690

C	0.65754910	3.79654993	-5.18301703

C	0.65282547	4.29968997	-3.92336018

C	1.92268118	4.12666535	-3.01720498

C	3.00818911	3.47626109	-3.50562534

C	4.24480486	1.30952146	-3.50438590

C	4.24314788	0.04410837	-3.02152529

C	3.76793214	-1.11979182	-3.90989611

C	3.34960496	-0.87870947	-5.17516655

C	2.10474269	-1.59838389	-5.72486862

C	1.33482714	-0.60418436	-6.61289009

C	-0.01943556	-0.60374114	-6.61252107

C	-0.78859050	0.72971156	-6.61211939

C	-1.69363362	2.92566500	-4.95084596

C	-2.45531267	1.58706174	-4.94369844

C	-2.03386976	0.56117356	-5.72286583

C	-2.03323366	-0.87661930	-5.17345578

C	-0.78891589	-1.59705799	-5.72372428

C	-0.11201429	-2.47226822	-4.94236713

C	1.42752109	-2.47299329	-4.94283443

C	1.90307347	-2.74683545	-3.50442216

C	2.99832030	-2.11309081	-3.02119340

C	3.77676627	2.47549831	-2.61476395

C	3.76729158	-0.22926816	-1.58321947

C	1.42702128	1.90270789	-6.61216334

B	-0.13230183	3.87705499	-1.54444567

BC59@Php

C	1.29350263	-2.47575679	0.29863477

C	1.11969089	-1.96665207	1.69526610

C	-0.04950414	-2.27721487	2.39040415

C	-1.14526173	-3.15190227	1.78448699

C	-0.98535824	-3.60831168	0.50565452

C	0.27675380	-3.22757685	-0.26146549

C	2.17059031	-1.48299086	-0.48948001

C	1.87167790	-0.62566171	1.80466777

C	1.07814706	0.33622879	2.53812953

C	-0.71307892	-1.23087326	3.23535407

C	-2.50621275	-2.64517911	2.25940511

C	-3.60060143	-2.64876972	1.38520961

C	-3.41708351	-3.16430576	-0.05020899

C	-2.16115625	-3.61549114	-0.46091043

C	-1.60984345	-3.22596169	-1.83130719

C	-0.09895607	-2.98907904	-1.68755476

C	0.57620211	-2.00684606	-2.39751504

C	1.69596969	-1.26598764	-1.85666038

C	2.27595507	1.04301652	-0.12010685

C	1.81007102	1.25162632	-1.49909492

C	1.71570793	0.11934640	-2.55505216

C	0.35585290	0.15425964	-3.34367061

C	-0.25074237	-1.10900900	-3.33899241

C	-2.35466214	-2.43218417	-2.67261555

C	-3.73228693	-1.94260007	-2.24340379

C	-4.24614270	-2.28040144	-0.98845296

C	-2.24540413	-1.44403338	3.16840324

C	-4.83149572	1.30679205	0.29237168

C	-4.46436290	1.09852861	1.63432058

C	-3.35582439	1.96288271	2.26678434

C	-2.72425777	2.95060972	1.51461445

C	-3.12427653	3.18029865	0.04970584

C	-3.92750640	1.86202803	-1.96628712

C	-4.51500611	0.44769815	-2.02798282

C	-5.08025619	0.09814913	-0.63309795

C	-4.94866086	-1.20697518	-0.13650581

C	-4.54722310	-1.43528689	1.33664622

C	-4.31207394	-0.33632323	2.17769744

C	-2.51193147	1.06075500	3.18473939

C	-1.11674142	1.23594173	3.24786698

C	-0.45191422	2.30446535	2.43456564

C	-1.20223747	3.12011638	1.61172895

C	-0.67115939	3.44179616	0.23437870

C	-1.84030096	3.48929438	-0.73523994

C	-1.66235465	3.00269260	-2.01928297

C	-2.74707027	2.15262858	-2.66607412

C	-3.86693222	-0.53618364	-2.78480229

C	-2.55635688	-0.20638249	-3.53974535

C	-2.03796176	1.06677635	-3.48814810

C	-0.52200602	1.24913708	-3.36210785

C	-0.29357080	2.43499919	-2.44407035

C	0.77088120	2.40786750	-1.55096624

C	0.55152959	2.91626832	-0.17037971

C	1.40318784	2.04727548	0.72296324

C	0.89829981	1.74451815	1.97883374

C	-3.10146446	-0.34878441	3.13794943

C	-0.15266489	0.03463223	3.27157489

C	-4.12424440	2.39930351	-0.53569042

C	-1.66549962	-1.30703756	-3.46262979

C	4.55173847	4.34404827	0.94183242

C	4.70793814	2.97211180	0.71753535

C	4.81883636	2.08555791	1.79864810

C	4.74273980	2.57094306	3.11153688

C	4.60774771	3.95079360	3.33676633

C	4.51410161	4.83542061	2.25211091

H	4.45889458	5.01568413	0.11277050

H	4.75013581	2.59719924	-0.28352052

H	4.79505976	1.89566204	3.93880116

H	4.57518763	4.32922818	4.33751427

H	4.41853278	5.88696820	2.42407219

C	5.01704050	0.72259789	1.53026144

C	6.38037175	0.28153114	2.07834990

C	3.95090768	0.35261046	2.55881630

C	6.37861969	-1.25798048	2.12825519

H	7.16523727	0.64211693	1.44665929

C	3.87983467	-1.17794508	2.62213961

H	4.21327874	0.74313212	3.51986158

C	5.25274459	-1.72343217	3.09030774

H	3.64812040	-1.56319719	1.65073807

H	5.22121032	-2.79258843	3.11417747

H	6.52185594	0.66788742	3.06817124

H	6.20541336	-1.64650205	1.14718876

H	5.45627818	-1.34749937	4.07067273

H	3.12187540	-1.47892176	3.31392735

H	3.00617996	0.75824079	2.26083697

N	4.56244262	-0.45435280	0.76750008

C	5.28461395	-0.57612816	-0.51603749

C	4.81347439	-1.75507973	1.43192061

C	4.87140615	-1.99423504	-0.91700304

H	6.34571156	-0.53089870	-0.37049473

C	5.30265404	-2.73813721	0.35111820

H	4.67885083	-1.97129405	2.47273681

H	5.33405699	-2.36684978	-1.80639002

H	4.89847474	-3.72773645	0.43092939

H	7.32307708	-1.61350849	2.48865327

B	3.01047839	-0.36060637	0.51609511

H	5.01347228	0.17996393	-1.22465747

H	3.81032702	-2.03776313	-1.05609551

H	6.36881695	-2.80943638	0.39233095

H	5.16598007	1.00421204	0.50723528

SiC59

C	-0.11472745	-0.55095891	0.09245325

C	1.42615096	-0.54776435	0.08854555

C	2.10521172	0.62738778	0.09233992

C	1.33817523	1.96636797	0.10790102

C	-0.03789419	1.96023747	0.12263805

C	-0.79695429	0.61914754	0.10412670

C	-0.58788866	-1.71331409	-0.80007172

C	0.65732904	-2.43107961	-1.35059994

C	1.90190237	-1.71082586	-0.80196511

C	2.99740023	-1.56207210	-1.58364969

C	3.35268362	0.79568898	-0.79917502

C	2.13275050	2.97084837	-0.78380909

C	1.47436799	3.86147527	-1.56014785

C	-0.83356169	2.95362485	-0.73804753

C	-2.06070239	2.23585664	-1.32081606

C	-2.04800187	0.78961006	-0.78860743

C	-2.45668231	-0.23185408	-1.57874055

C	-1.68400598	-1.56396160	-1.58224934

C	0.65770364	-2.91564142	-2.61555344

C	-0.58753008	-2.74597102	-3.50413896

C	-1.68264463	-2.11224478	-3.02037993

C	-2.45137066	-1.11766721	-3.90841388

C	-2.92873686	0.04561429	-3.01905553

C	-2.93256105	1.31381328	-3.50256046

C	-2.47117599	2.48541411	-2.61041081

C	-1.70460757	3.49371009	-3.52210643

C	-0.61702333	4.14643957	-3.05254228

C	3.36269851	2.23671031	-1.34270162

C	1.90312874	3.06924511	-5.72599279

C	3.00068244	2.91969745	-4.94279040

C	3.76758743	1.58379211	-4.94196054

C	3.34885287	0.55970067	-5.72358467

C	2.10393827	0.72932746	-6.61194244

C	-0.11232297	1.90320542	-6.61358568

C	-0.59072253	3.07059350	-5.73135690

C	0.65754910	3.79654993	-5.18301703

C	0.65282547	4.29968997	-3.92336018

C	1.92268118	4.12666535	-3.01720498

C	3.00818911	3.47626109	-3.50562534

C	4.24480486	1.30952146	-3.50438590

C	4.24314788	0.04410837	-3.02152529

C	3.76793214	-1.11979182	-3.90989611

C	3.34960496	-0.87870947	-5.17516655

C	2.10474269	-1.59838389	-5.72486862

C	1.33482714	-0.60418436	-6.61289009

C	-0.01943556	-0.60374114	-6.61252107

C	-0.78859050	0.72971156	-6.61211939

C	-1.69363362	2.92566500	-4.95084596

C	-2.45531267	1.58706174	-4.94369844

C	-2.03386976	0.56117356	-5.72286583

C	-2.03323366	-0.87661930	-5.17345578

C	-0.78891589	-1.59705799	-5.72372428

C	-0.11201429	-2.47226822	-4.94236713

C	1.42752109	-2.47299329	-4.94283443

C	1.90307347	-2.74683545	-3.50442216

C	2.99832030	-2.11309081	-3.02119340

C	3.77676627	2.47549831	-2.61476395

C	3.76729158	-0.22926816	-1.58321947

C	1.42702128	1.90270789	-6.61216334

Si	-0.13230183	3.87705499	-1.54444567

SiC59@Php

C	1.29350263	-2.47575679	0.29863477

C	1.11969089	-1.96665207	1.69526610

C	-0.04950414	-2.27721487	2.39040415

C	-1.14526173	-3.15190227	1.78448699

C	-0.98535824	-3.60831168	0.50565452

C	0.27675380	-3.22757685	-0.26146549

C	2.17059031	-1.48299086	-0.48948001

C	1.87167790	-0.62566171	1.80466777

C	1.07814706	0.33622879	2.53812953

C	-0.71307892	-1.23087326	3.23535407

C	-2.50621275	-2.64517911	2.25940511

C	-3.60060143	-2.64876972	1.38520961

C	-3.41708351	-3.16430576	-0.05020899

C	-2.16115625	-3.61549114	-0.46091043

C	-1.60984345	-3.22596169	-1.83130719

C	-0.09895607	-2.98907904	-1.68755476

C	0.57620211	-2.00684606	-2.39751504

C	1.69596969	-1.26598764	-1.85666038

C	2.27595507	1.04301652	-0.12010685

C	1.81007102	1.25162632	-1.49909492

C	1.71570793	0.11934640	-2.55505216

C	0.35585290	0.15425964	-3.34367061

C	-0.25074237	-1.10900900	-3.33899241

C	-2.35466214	-2.43218417	-2.67261555

C	-3.73228693	-1.94260007	-2.24340379

C	-4.24614270	-2.28040144	-0.98845296

C	-2.24540413	-1.44403338	3.16840324

C	-4.83149572	1.30679205	0.29237168

C	-4.46436290	1.09852861	1.63432058

C	-3.35582439	1.96288271	2.26678434

C	-2.72425777	2.95060972	1.51461445

C	-3.12427653	3.18029865	0.04970584

C	-3.92750640	1.86202803	-1.96628712

C	-4.51500611	0.44769815	-2.02798282

C	-5.08025619	0.09814913	-0.63309795

C	-4.94866086	-1.20697518	-0.13650581

C	-4.54722310	-1.43528689	1.33664622

C	-4.31207394	-0.33632323	2.17769744

C	-2.51193147	1.06075500	3.18473939

C	-1.11674142	1.23594173	3.24786698

C	-0.45191422	2.30446535	2.43456564

C	-1.20223747	3.12011638	1.61172895

C	-0.67115939	3.44179616	0.23437870

C	-1.84030096	3.48929438	-0.73523994

C	-1.66235465	3.00269260	-2.01928297

C	-2.74707027	2.15262858	-2.66607412

C	-3.86693222	-0.53618364	-2.78480229

C	-2.55635688	-0.20638249	-3.53974535

C	-2.03796176	1.06677635	-3.48814810

C	-0.52200602	1.24913708	-3.36210785

C	-0.29357080	2.43499919	-2.44407035

C	0.77088120	2.40786750	-1.55096624

C	0.55152959	2.91626832	-0.17037971

C	1.40318784	2.04727548	0.72296324

C	0.89829981	1.74451815	1.97883374

C	-3.10146446	-0.34878441	3.13794943

C	-0.15266489	0.03463223	3.27157489

C	-4.12424440	2.39930351	-0.53569042

C	-1.66549962	-1.30703756	-3.46262979

C	4.55173847	4.34404827	0.94183242

C	4.70793814	2.97211180	0.71753535

C	4.81883636	2.08555791	1.79864810

C	4.74273980	2.57094306	3.11153688

C	4.60774771	3.95079360	3.33676633

C	4.51410161	4.83542061	2.25211091

H	4.45889458	5.01568413	0.11277050

H	4.75013581	2.59719924	-0.28352052

H	4.79505976	1.89566204	3.93880116

H	4.57518763	4.32922818	4.33751427

H	4.41853278	5.88696820	2.42407219

C	5.01704050	0.72259789	1.53026144

C	6.38037175	0.28153114	2.07834990

C	3.95090768	0.35261046	2.55881630

C	6.37861969	-1.25798048	2.12825519

H	7.16523727	0.64211693	1.44665929

C	3.87983467	-1.17794508	2.62213961

H	4.21327874	0.74313212	3.51986158

C	5.25274459	-1.72343217	3.09030774

H	3.64812040	-1.56319719	1.65073807

H	5.22121032	-2.79258843	3.11417747

H	6.52185594	0.66788742	3.06817124

H	6.20541336	-1.64650205	1.14718876

H	5.45627818	-1.34749937	4.07067273

H	3.12187540	-1.47892176	3.31392735

H	3.00617996	0.75824079	2.26083697

N	4.56244262	-0.45435280	0.76750008

C	5.28461395	-0.57612816	-0.51603749

C	4.81347439	-1.75507973	1.43192061

C	4.87140615	-1.99423504	-0.91700304

H	6.34571156	-0.53089870	-0.37049473

C	5.30265404	-2.73813721	0.35111820

H	4.67885083	-1.97129405	2.47273681

H	5.33405699	-2.36684978	-1.80639002

H	4.89847474	-3.72773645	0.43092939

H	7.32307708	-1.61350849	2.48865327

Si	3.01047839	-0.36060637	0.51609511

H	5.01347228	0.17996393	-1.22465747

H	3.81032702	-2.03776313	-1.05609551

H	6.36881695	-2.80943638	0.39233095

H	5.16598007	1.00421204	0.50723528

The optimized energy profiles for all investigated structures provide clear evidence of stable, reliable convergence with both the B97D and ωB97XD functionals. In all cases, the total energy decreases significantly during the initial optimization steps, indicating rapid structural relaxation from the initial geometry toward lower-energy configurations. This is followed by a gradual leveling off of the energy curves, confirming that each system successfully reaches a minimum-energy state. For pristine structures (C_60_, BC_59_, and SiC_59_), the convergence behavior is smooth and monotonic under both functionals, with no significant oscillations, reflecting straightforward optimization pathways and structurally stable frameworks. In contrast, the adsorbed complexes (C_60_@Php, BC_59_@Php, and SiC_59_@Php) exhibit slightly more complex behavior, particularly in the early stages of optimization. Despite these intermediate variations, all systems ultimately reach well-defined energy plateaus, confirming successful convergence. These validation results indicate that the optimized geometries can be reliable for subsequent electronic, absorption, and sensing analyses (Figure S1).

Fig S1. Optimization energy profiles of pristine (C_60_, BC_59_, and SiC_59_) and adsorbed (C_60_@Php, BC_59_@Php, and SiC_59_@Php) systems as a function of optimization steps calculated using B97D and ωB97XD functionals. The rapid initial decrease in energy followed by stable plateaus confirms efficient convergence toward minimum energy geometries for all structures.

Rather than repeating the numerical HLG discussion, the DOS profiles mainly show how B/Si doping and Php adsorption redistribute the electronic states near the frontier region, particularly for BC_59_@Php. Figure S2 supports the HLG trend shown in Table 2. Pristine C_60_ exhibits a wider frontier-orbital separation, whereas B/Si substitution and Php adsorption shift the electronic states toward the frontier region. The most evident redistribution is observed for BC_59_@Php, consistent with its stronger adsorption-induced electronic response.

Fig S2. DOS maps for C_60_ and its derivatives in the presence/absence of Php.

The pristine C_60_, BC_59_, and SiC_59_ structures exhibit comparable PDOS patterns, with changes in peak positions and intensities caused by B and Si doping. After Php adsorption, new molecular contributions appear and overlap with the C_60_ states, modifying the total DOS near the frontier-energy region. The observed orbital mixing suggests electronic coupling and charge redistribution between Php and the fullerene surfaces (Fig S3).

Fig S3. Calculated TDOS and PDOS spectra of pristine and doped fullerenes (C60, BC59, and SiC59) before and after Php adsorption.

To examine the effect of the attempt-frequency treatment, the B97D/6-31G(d) recovery times were also recalculated using system-specific frequencies extracted from the harmonic vibrational outputs. The resulting τvib values were 8.79×10^-8^, 6.98×10^-7^, and 9.86×10^15^ s for C60@Php, BC59@Php, and SiC59@Php, respectively, compared with the corresponding fixed-prefactor values of 1.28×10^-7^, 7.97×10^-7^, and 2.81×10^16^ s. The absolute differences between the corresponding log_10_(τ) values were 0.16, 0.06, and 0.45, respectively. Therefore, the two treatments show close agreement on the logarithmic scale, with all differences remaining below 0.5 logarithmic units.

Importantly, both prefactor treatments preserve the same recovery-time ordering, C60@Php < BC59@Php ≪ SiC59@Php. Both approaches predict rapid desorption for C60@Php and BC59@Php but exceptionally slow predicted desorption for SiC59@Php. Thus, replacing the conventional fixed prefactor with the vibrationally derived values changes the absolute recovery-time estimates by factors of approximately 1.46, 1.14, and 2.85, respectively, without affecting the principal interpretation or structural ranking (Table S2).

| Table S2. Comparison of recovery-time estimates for the C60@Php, BC59@Php, and SiC59@Php complexes. Here, νdes is the selected desorption-related intermolecular vibrational wavenumber in cm^-1^; ν_0_,_vib_ is the corresponding system-specific vibrational attempt frequency in s^-1^; τ_fixed_ is the recovery time calculated using the fixed attempt frequency ν_0_=10^12^ s^-1^; log_10_(τ_fixed_) is its decimal logarithm; τ_vib_ is the recovery time calculated using ν_0_,vib; and log_10_(τ_vib_) is its decimal logarithm. | | | | | | |
| --- | --- | --- | --- | --- | --- | --- |
| Structure | ${\tilde{\boldsymbol{\nu}}}_{\boldsymbol{des}}\boldsymbol{(}\boldsymbol{cm}^{\boldsymbol{-}\boldsymbol{1}}\boldsymbol{)}$ | $\boldsymbol{\nu}_{\boldsymbol{0}\boldsymbol{,}\boldsymbol{vib}}\boldsymbol{(}\boldsymbol{s}^{\boldsymbol{-}\boldsymbol{1}}\boldsymbol{)}$ | $\boldsymbol{\tau}_{\boldsymbol{fixed}}\boldsymbol{(}\boldsymbol{s}\boldsymbol{)}$ | $\boldsymbol{Log}_{\boldsymbol{10}}\boldsymbol{(}\boldsymbol{\tau}_{\boldsymbol{fixed}}\boldsymbol{)}$ | $\boldsymbol{\tau}_{\boldsymbol{vib}}\boldsymbol{(}\boldsymbol{s}\boldsymbol{)}$ | $\boldsymbol{Log}_{\boldsymbol{10}}\boldsymbol{(}\boldsymbol{\tau}_{\boldsymbol{vib}}\boldsymbol{)}$ |
| C60@Php | 48.5883 | 1.4566×10^12^ | 1.28×10^-7^ | -6.88 | 8.79×10^-8^ | -7.06 |
| BC59@Php | 38.0897 | 1.1419×10^12^ | 7.97×10^-7^ | -6.09 | 6.98×10^-7^ | -6.16 |
| SiC59@Php | 95.0201 | 2.8486×10^12^ | 2.81×10^16^ | 16.44 | 9.86×10^15^ | 15.99 |
